# Insights and Recommendations From Moderators and Community Members for Keeping Online Peer Support Safe: Thematic Analysis

**DOI:** 10.2196/81943

**Published:** 2026-03-12

**Authors:** Hannah Grace Jones, Grace Lavelle, Elly Aylwin-Foster, Ciara Regan, Alan Simpson, Ewan Carr, Matthew Hotopf, Vanessa Lawrence

**Affiliations:** 1 Department of Psychological Medicine, Institute of Psychiatry, Psychology and Neuroscience, King's College London London United Kingdom; 2 Lifelong Health Persistent Pain Research Group, Hopwood Centre for Neurobiology South Australian Health and Medical Research Institute Adelaide, South Australia Australia; 3 IIMPACT (Innovation, IMPlementation and Clinical Translation) in Health School of Allied Health and Human Performance Adelaide University Adelaide, South Australia Australia; 4 Health Service and Population Research, Institute of Psychiatry, Psychology and Neuroscience, King’s College London London United Kingdom; 5 Florence Nightingale Faculty of Nursing, Midwifery & Palliative Care, King’s College London, United Kingdom London United Kingdom; 6 Department of Biostatistics & Health Informatics, Institute of Psychiatry, Psychology and Neuroscience, King’s College London, United Kingdom London United Kingdom

**Keywords:** chronic conditions, depression, long-term physical health conditions, mental wellbeing, moderation, online peer support, online safety

## Abstract

**Background:**

Online peer support can help people living with long-term physical health conditions to manage their mental well-being. Although the potential negative events that can occur and risks associated with web-based peer communities are well recognized, our understanding of how best to moderate these spaces is relatively limited, particularly with regard to new communities. Previous work has focused on the experiences of either moderators or community members.

**Objective:**

This study aims to explore the perspectives of both members and moderators of a new online peer support community to evaluate the moderation procedures and inform recommendations for best practice.

**Methods:**

Community members (n=39) who participated in a research trial of a new online peer community, CommonGround, were interviewed. The moderation team (n=5) was invited to a focus group. Community member interviews explored their opinions of moderation policies and the behavior of the moderation team. The moderator focus group explored their experiences of moderating the community, including perceived benefits, common challenges, and areas for improvement. All interviews and the focus group were conducted online, audio-recorded, and transcribed verbatim. An inductive thematic analysis was conducted to sort the data into overarching themes through an iterative process.

**Results:**

Effective moderation was considered critical in creating a safe space that members wanted to engage with and for mitigating any risks, particularly around the spread of medical misinformation. Both moderators and community members felt that the moderation policies and practices were appropriate and applicable to the community. Moderators found navigating the moderation threshold, where they balanced safety against free speech, challenging when determining whether to intervene or not. Being part of a team with mixed clinical expertise helped moderators build confidence in navigating this threshold and also presented other benefits of easy access to support and improving the consistency of their moderation practices. It was suggested that in order for a community to flourish, community members would self-moderate. However, moderators and members felt that the strong community culture and high levels of member engagement that are needed to support self-moderation had not yet evolved. Proposed improvements to moderation included new features to support the efficiency of identifying new content for review and reviewing the rule of anonymity.

**Conclusions:**

Moderation is critical in making online peer communities feel safe and engaging. Moderation practices should be co-produced with the target audience to ensure that they are aligned with the community’s unique moderation wants and needs, including clear escalation pathways, transparent communication patterns, and plans to review and update policies or procedures as the community evolves. There should be technological features that promote self-moderation, as the community may shift towards self-moderation as it matures. It is also critical to ensure that moderators feel supported so that they are best placed to support the broader community.

**Trial Registration:**

ClinicalTrials.gov NCT06222346; https://clinicaltrials.gov/study/NCT06222346

**International Registered Report Identifier (IRRID):**

RR2-10.2196/71513

## Introduction

Connecting with peers or other people “like me” who have similar life experiences can be a powerful way of receiving social support and managing one’s mental well-being [1,2]. By engaging with peers, people can exchange emotional support and their experiential knowledge [3]. In the last 10 years, there has been a rise in the use of online peer support, with the web-based setting facilitating convenient and flexible engagement that overcomes any physical barriers to accessing in-person support groups [4]. In particular, asynchronous peer community forums have become popular, as they provide the opportunity to exchange support with a potentially unlimited number of peers around the clock [5,6]. Those living with mental health conditions who have engaged with web-based peer support have experienced improved mental health symptoms, quality of life, and medical self-management skills [2,7,8]. Web-based peer support, therefore, offers a highly scalable, low-cost intervention that has the potential to promote mental well-being and reduce the burden of negative mental health on the individual, society, and health care system [4,9].

One population who may particularly benefit from online peer support is people living with long-term physical health conditions, as this population often reports experiencing psychological distress, loneliness, and disconnection from people in their social circles [10-12]. Around 15 million people in the United Kingdom live with a long-term physical health condition [13]. Of these, approximately 3 million are also living with symptoms of low mood that negatively impact their physical health [14], hinder effective self-management, and impair quality of life [15,16]. These symptoms of low mood also put them at risk of developing major depressive disorder, highlighting the critical need to intervene early to mitigate the impacts of mental-physical comorbidities on individuals, families, society, and health services [17-19]. Despite initial evidence documenting the psychosocial benefits of engaging with online peer support for people with long-term conditions [1,20-24], the provision of peer support specifically tailored to the needs of this population is relatively limited [25,26]. Our own qualitative research with people with diverse long-term physical health conditions found that traditional condition-specific communities can limit their ability to connect on other commonalities beyond their diagnosis and may not be appropriate for those with rare or multiple conditions [27,28]. To address this unmet need, we worked alongside people living with long-term health conditions, clinicians, and software developers in an iterative coproduction process to create CommonGround: an online peer support and psychoeducation platform that aims to intervene early to prevent the progression of symptoms of low mood to major depressive disorder [29]. By placing the voices of people with long-term health conditions in the foreground, we have tailored the intervention design and features specifically to their needs, including the all-diagnoses-welcome ethos that reflects the view that many experiences and challenges transcend diagnoses.

While online peer support communities have the potential to enhance the well-being of their members, there are challenges and risks associated with them. People with long-term health conditions have shared concerns about the potential negative outcomes from engaging digitally with their peers and the need for web-based spaces to feel safe [27,30,31]. Web-based forums carry inherent risks of medical misinformation and the sharing or encouragement of risky and unsafe behaviors [9,31]. Hearing about peers’ experiences can potentially exacerbate low mood and promote symptom-comparisons that generate anxiety about one’s condition [27,32-34]. Behind the screen of anonymity, people can argue, have hostile interactions, and experience cyberbullying or trolling [35,36]. It is well acknowledged that appropriate community governance is essential in reducing the risk of these negative outcomes arising, particularly among communities of people with long-term health conditions who are likely to discuss sensitive, personal, and medical-related topics.

One means of mitigating the risks of online peer support is through moderation by professional moderators. Clear, consistent, and comprehensive moderation policies play an essential role in transparently curating trust and safety in the community. By clearly outlining boundaries and acceptable behavior, community members can make judgments on whether the community is a space they wish to attend and have a clear understanding of how to behave within the community. Professional moderators can play an essential role in implementing these policies, including identifying and removing inappropriate content, preventing conflicts between members, enforcing the community guidelines, and supporting those in potential crisis [37-39]. Often, these moderators consider themselves as a safety net and draw on their expertise to identify and eliminate hazardous scenarios that could cause harm, such as the sharing of unsafe or fraudulent medical advice and promotions of self-harm or suicide [9,37]. In addition to their safeguarding role, moderators can help establish a supportive, thriving community by creating a safe space for personal disclosures and fostering positive relations between members [40]. Indeed, moderated communities have been found to have higher engagement, perceived social support, and acceptability to members than unmoderated communities [41]. The impact of moderators within a community has also been noted in real-world settings where shifts in community moderation happen. For instance, following the departure of moderators from an established web-based health community, community members felt reluctant to engage with the community and recognized that the professional moderators had played a unique role in fairly and consistently implementing the rules and regulations [31]. Appropriate moderation is therefore often considered essential in laying the foundations of safety and facilitating the success of web-based communities. In the creation of CommonGround, the voices of lived experience experts were central to determining the Community Principles (“terms of use” community members agree to) and the associated governing policies to ensure that they remained aligned with their expectations of moderation and priorities for safety.

Much work has focused on how specific moderation issues are handled in well-established communities [9,30,31], yet there is limited guidance or recommendations on how to integrate moderation practices into a newly founded peer support community [37]. In addition, even though their experiences are inextricably intertwined, many studies consider the experiences of either the moderator [9,30] or the community member in isolation [31], and do not synthesize these perspectives to create a comprehensive understanding of what best practice might look like. Whilst moderators and community members participate with communities for different reasons, they coexist in the same space and their experiences are consistently shaped by the presence and behavior of one another. Moderators help establish the community culture and the boundaries for participation, while the behavior and responses of community members, in turn, influence what moderators do (eg, the content they have to moderate) and how they adapt their moderation responsively to suit the needs of the community. In this way, the experiences and behaviors of moderators and communities are continually shaping one another over time and cannot occur independently of one another. We therefore explored moderation experiences within the newly formed CommonGround community from the perspective of both community members and moderators. Our objective was to evaluate the feasibility and acceptability of the moderation procedures and inform recommendations for best practice in moderating newly forming web-based peer support communities.

## Methods

### Design

This work exists within the wider context of the CommonGround project, which aimed to coproduce an online peer support and psychoeducation platform and assess the feasibility and acceptability of the intervention in a randomized controlled trial (fRCT [feasibility randomized controlled trial] [42]; ClinicalTrials.gov: NCT06222346). The CommonGround project is underpinned by a pragmatic mixed methods research paradigm with community participation, which prioritizes what works, for whom, and in what context. The fRCT was delivered remotely and randomized 83 participants with long-term physical health conditions and subthreshold depression to the CommonGround intervention (ie, community members) and 42 participants to the control condition of National Health Service Mental Health webpages for 3 months (findings published in full elsewhere; in press). A group of 187 people with long-term physical health conditions who were interested in managing their mental health better, but did not have subthreshold depression, were also onboarded onto the CommonGround platform to increase the size of the community (community engagement cohort [29]). A nested qualitative process evaluation examined the experiences of community members with long-term physical health conditions and moderators. The qualitative process evaluation adopts an interpretivist-constructivist approach that assumes realities are socially constructed through interactions with others in the cultural context in which they are situated. This paper is reported within the SRQR (Standards for Reporting Qualitative Research) guidelines as per EQUATOR (Enhancing the Quality and Transparency of Health Research) recommendations (Multimedia Appendix 1 [43]).

### Ethical Considerations

This study involves data from the nested qualitative study of part of a larger feasibility randomized control trial that was conducted remotely at King’s College London (KCL). The trial and nested qualitative study received ethical approval from the South Central - Oxford C Research Ethics Committee (IRAS ID 328175). The protocol was preregistered (ClinicalTrials.gov: NCT06222346) [29] and outlined the larger feasibility randomized controlled trial and the nested qualitative study (ie, community member and control group interviews, moderator focus group). This project is sponsored and organized by King’s College London. The Research Governance Office can be contacted via email at rgo@kcl.ac.uk.

The data from the nested qualitative study were collected in alignment with the original protocol aims of understanding participant perspectives of the intervention, control condition, and trial procedures generally (published elsewhere [29]) and for understanding perspectives and experiences of moderation specifically (this study).

Informed consent was obtained from all community members at two stages: (1) at T_1_ when completing the 2 eligibility screenings, and if found to be eligible for the trial, (2) at T_2_ when they consented to participate in the trial. All community members had provided consent to take part in the feasibility randomized controlled trial at enrolment. At enrolment, they were informed that they may be invited to interview after the 3-month intervention period, and that additional consent would be sought at that time if selected. Consent was recapped for those who were invited to interview prior to their agreement to interview.

Community members and moderators were invited to be interviewed or take part in the focus group, respectively, after the intervention period. Five days after the invite, they were contacted again to discuss any questions about what taking part would involve before completing a web-based consent form (via Qualtrics) and scheduling the interview. Participants were allowed more time to consider their participation if needed and asked to contact the research team should they decide they wished to participate.

At the beginning of the interviews and focus group, the information about what the session would involve was repeated verbally, with participants reminded of their right to withdraw and to decline to answer any question, at any time. Community members were reimbursed £20 (US $26.93) for their participation, and moderators were reimbursed in alignment with their hourly wage.

### The CommonGround Platform

CommonGround was codesigned in collaboration with people with lived experience of long-term conditions or physical-mental health comorbidities, clinicians, academics, and software developers [28]. The anonymous web-based platform was open to all diagnoses and could be accessed via desktop or mobile devices. The key features included a discussion forum with different posting options, reactions, and the ability to comment (peer support component), alongside a resources page of evidence-based self-help information (psychoeducation component). The self-help information consisted of 91 resources that were hand-selected by the research team from reliable, reputable sources and covered various topics ranging from keeping active, managing mental well-being, and managing finances. The resources were selected for their relevance to people living with any long-term physical health condition (ie, were not condition-specific). The CommonGround platform features, functions, and layout remained static throughout the intervention period with one minor exception. During the first week, the software developers made a minor edit to how the latest activity in the community forum appeared. Initially, at launch, the newest post was displayed at the top of the community forum feed. This was changed so that the post with the most recent activity displayed at the top of the feed (ie, existing post with the most recent comment or new post). Further details of the features of CommonGround and screenshots of the platform are available in Multimedia Appendix 2.

### Moderation Policies and Practices

The CommonGround platform was governed by the Community Principles, Moderation Policy, Safeguarding Policy, and Privacy Policy, which were all published on the platform (and are available on the CommonGround projects Open Science Framework webpage [44]). These policies were coproduced in collaboration with our research advisory group (10 members living with a range of long-term health conditions, including 6 females and 4 males (self-reported sex), aged between 25 and 73 years), software developers, and the Samaritans Online Harms Team [45]. Key principles of the moderation approach included that the platform was anonymous, was not moderated 24/7, and did not provide one-to-one or crisis support. The moderation team also had access to a moderator handbook, developed in consultation with a leading disability charity [46], which included guidance on how to moderate different scenarios. The CommonGround platform had an admin backend that allowed moderators to edit and delete posts, delete comments, review flagged posts, and escalate posts for further action. Community members were able to view all policies and “flag” posts for review by moderators. Prior to the launch of the community, moderators were trained by the research team on how to use CommonGround and how to implement the governing policies. The moderator training was developed based on consultations with the community manager at SCOPE [47]. Each moderation session was expected to take approximately 60 minutes per day, and to occur at some time between the hours of 8 AM and 8 PM. Moderators were able to choose whether to moderate across a single or multiple sessions, and exactly when to moderate, dependent on the needs of the community (eg, when there was a moderation concern to monitor) and their personal schedules. Moderators were required to complete a handover after each moderation session of outstanding tasks, posts, or comments to watch, and any other comments. Moderators were instructed to review the previous moderation handover at the beginning of their session and to complete any outstanding tasks as a priority. Then moderators were instructed to review new content and complete any moderation tasks as needed (ie, in response to what had been posted). These moderation tasks may have involved any (or all) of the following: edit posts, delete posts, delete comments, review posts with attachments, review flagged posts, comment on posts to guide discussions toward a productive versus harmful narrative, and contact users to issue warnings. A Microsoft Teams chat was also created to support shared decision-making and provide updates to the moderation team. All moderators were of equal status and consulted with the research team (HGJ, GL, and MH) as required for additional support or to resolve any queries they had on the best course of action. For instance, if a moderator was unsure about whether to moderate or not (eg, whether to delete a comment, an action that could not be reversed), they were encouraged to discuss this with the team to come to a collective decision. If required, any decisions to issue warnings to community members (part of the “three strike” policy) or to remove community members from CommonGround would be made by joint decision of the moderators and research team. At any time, moderators could request an informal debrief with the research team, which included a liaison psychiatrist. A feedback session for moderators to share experiences was conducted halfway through the 3-month intervention period.

There was a separate engagement team whose exclusive focus was on cultivating engagement on CommonGround by sharing discussion topics to stimulate conversation and weekly circular emails with community updates and reminders to log in. The engagement team had no moderating or safeguarding responsibilities, but could use the “flag this post” button if they came across something they felt required attention (as a regular community member could also do). The engagement team consisted of our co-researcher with lived experience and 2 other research team members. The profiles of the Engagement Team on the platform were clearly identifiable as being distinct from both regular community members and the Moderation Team via their avatar and account username. As the engagement team involved different team members to the moderation team, had unique roles and responsibilities, and were not involved in any moderation activity, the engagement team will not be discussed further in this manuscript.

### Participants

#### CommonGround Community Members

CommonGround community members were recruited in the United Kingdom through multiple channels, including outpatient clinics within King’s College Hospital and Guy’s and St Thomas’s Hospital, public advertisements via charities, and word-of-mouth. The inclusion/exclusion criteria for the feasibility trial are listed in Table 1 and were all self-reported by participants. As mentioned above, to increase the size of the community at the time that CommonGround was launched, people who did not have subthreshold depression (ie, scored 0-4 or 10-14 on 8-item Patient Health Questionnaire) but met all remaining inclusion criteria were also offered the opportunity to join CommonGround as part of a community cohort study (study protocol contains full details [29]). The only difference between participants of the feasibility trial and the community cohort was their 8-item Patient Health Questionnaire scores: feasibility trial participants scored 5-9, whereas community cohort scored either 0-4 or 10-14. Approximately 40 community members who had access to CommonGround (including ≈30 fRCT participants and ≈10 community engagement cohort) were invited to participate in an interview via email with a follow-up phone call. Community members were approached purposefully based on their long-term condition(s), age, gender, ethnicity, and their engagement level with the community. The engagement levels were defined as follows: (1) never joined (never registered their account or logged in), (2) never logged in (created an account but never logged in), (3) readers (logged in but never commented, reacted, or posted), (4) acknowledgers (logged in and commented or reacted, but did not post), and (5) content creators (logged in and created posts).

**Table 1:** Inclusion and exclusion criteria for the feasibility randomized controlled trial (fRCT) and community cohort.

	Inclusion criteria	Exclusion criteria
fRCT	Currently living with one or more long-term conditions. A long-term condition was defined as a diagnosed physical medical condition with a duration of ≥6 months that demonstrates recurrence or deterioration and is typically associated with a poor prognosis [48]. There were no restrictions on the type of long-term condition. Probable subthreshold depression, defined as scoring 5-9 on the PHQ-8^a^. Age ≥18 years. Have access to the internet (via phone, smart device, desktop, or laptop at any location, including at home, at the workplace, or free Wi-Fi in public spaces). Have sufficient English proficiency to engage with the platform. Ability to give informed consent (ie, no known circumstances/conditions that may affect the capacity to consent).	Has ever received a clinical diagnosis of severe mental illness, including bipolar disorder, psychosis, posttraumatic stress disorder (PTSD), and schizophrenia. Has ever received a clinical diagnosis of dementia.
Community cohort	Currently living with one or more long-term conditions. A long-term condition was defined as a diagnosed physical medical condition with a duration of ≥6 months that demonstrates recurrence or deterioration and is typically associated with a poor prognosis [48]. There were no restrictions on the type of long-term condition. Scoring 0-4 or 10-14 on the PHQ-8a. Age ≥18 years. Have access to the internet (via phone, smart device, desktop, or laptop at any location, including at home, at the workplace, or free Wi-Fi in public spaces). Have sufficient English proficiency to engage with the platform. Ability to give informed consent (ie, no known circumstances/conditions that may affect the capacity to consent).	Has ever received a clinical diagnosis of severe mental illness, including bipolar disorder, psychosis, PTSD, and schizophrenia. Has ever received a clinical diagnosis of dementia.

^a^PHQ-8: 8-item Patient Health Questionnaire.

#### Moderators

Moderators were recruited through KCL PhD cohorts and through professional connections of the research team, based on their clinical expertise in mental health treatment and experience of attending or moderating peer support groups. At the end of the trial period, all moderators (n=5) were invited via email to participate in a focus group.

### Data Collection

#### Interviews

Demographic data were collected at baseline as part of the fRCT via anonymous Qualtrics questionnaires. Interviews were conducted after the 3-month intervention period (September 28, 2024, to December 8, 2024) by a member of the research team (CR, HGJ, GL) to explore individual experiences of taking part in the trial and of the CommonGround intervention. Topic guides were coproduced with the research advisory group (Multimedia Appendix 3). The topic guides included the following open-ended questions around moderation: “What were your experiences, if any, of the Moderation Team on CommonGround?” and “What did you think about CommonGround being an anonymous platform?” All interviews lasted approximately 30-90 minutes and were conducted online via Microsoft Teams and audio-recorded, before being transcribed verbatim and anonymized for analysis.

#### Focus Group

Demographic data were collected via Qualtrics. A focus group was conducted (by a member of the research team, CR, who had no prior contact with the moderators during the trial) to stimulate debate and discussion around their experiences of moderating the platform, moderation policies and training, the positive and negative experiences they had observed community members have, and how moderation could be improved in the future. Moderators were given the opportunity to elaborate on any topic they felt was relevant that may not have been covered by the topic guides and were prompted to provide specific examples. The focus group was conducted via Microsoft Teams, audio-recorded, and transcribed verbatim and anonymized for analysis. The focus group lasted for approximately 90 minutes.

### Data Analysis

Data from the community members (interviews) and moderators (focus group) were analyzed together using NVivo software (version 14, released 2023; Lumivero). An inductive thematic analysis was performed to sort the data into overarching themes that were developed inductively and from an interpretivist-constructivist position [49,50]. The first author (HGJ) familiarized herself with the entire dataset by reading and rereading the transcripts. Once immersed in the data, an initial list of codes was generated, which were constantly compared with one another to identify patterns of shared meaning that formed provisional themes. An experienced qualitative researcher (VL) reviewed the initial coding framework to help refine the codes and provide an additional perspective on interpreting the data. VL and HGJ also met regularly to discuss the analysis and explore different interpretations. The transcripts were then reviewed, and relevant extracts of the data were coded into NVivo nodes (the refined codes). Through an iterative process, codes were refined, added, and condensed as needed before being organized into themes. Following further discussions, a consensus was reached on the analysis, and the themes were named and defined. Throughout, our lived experience co-researcher (EA-F) ensured the coding framework was reflective of lived experience terminologies and definitions.

## Results

### Participant Characteristics

#### Community Members

A total of 39 participants who had been provided access to the CommonGround intervention were interviewed (29 fRCT participants, 10 Community Engagement participants), including 21 women, including transgender women, and 18 men, including transgender men, living with a wide variety of long-term health conditions (Table 2). Data saturation was reached after the 39 interviews, and no further interviews were conducted.

**Table 2:** Demographics of the community members interviewed.

Category		Overall (n=39)
Age (years), mean (SD)		53.2 (13.7)
**Gender (n=39), n (%)**		
	Women, including transgender women	21 (53.8)
	Men, including transgender men	18 (46.2)
**Ethnicity, n (%)**		
	Asian or Asian British	—^a^
	Black, Black British, Caribbean or African	—
	Other ethnic group	—
	Prefer not to say	—
	White	31 (79.5)
	Mixed or multiple ethnic groups	—
**Engagement level with CommonGround (n=39), n (%)**		
	Never joined: never registered their account or logged in	6 (15.4)
	Never logged in: created an account but never logged in	5 (12.8)
	Readers: logged in but never comments, reacted, or posted	5 (12.8)
	Acknowledgers: logged in and commented or reacted, but did not post	7 (17.9)
	Content creators: logged in and created posts	16 (41.1)
**Long-term physical health conditions: total, n**		
	Total number of unique LTCs^b^ represented	31
**Long-term physical health conditions: categories, n**		
	Autoimmune and inflammatory	11
	Cancer	—
	Cardiovascular	9
	Gastrointestinal and digestive	18
	Metabolic and endocrine	6
	Musculoskeletal and chronic pain	9
	Neurological (eg, Parkinsons)	13
	Other	8
	Reproductive and gynecological	—
	Respiratory	—

^a^Represents <5 participants, removed to protect anonymity.

^b^LTC: long-term condition.

#### Moderators

The moderation team included 4 women and 1 man (self-reported gender), aged between 26 and 45 years. Three were clinical research fellows. One was a mental health nurse, one a pharmacist, one a clinical psychologist, and 2 were adult psychiatrists. Four had clinical experience in working with people with mental and physical health conditions, and one had experience working with people with mental health conditions only. Their years of clinical experience ranged from 6 to 19 years (mean 10, SD 5.1 years). All the moderators were currently completing PhDs.

### Themes and Subthemes

#### Overview

Data from both groups of participants converged around five key themes: (1) moderation as a pillar of community safety, (2) the moderation blueprint, (3) the moderation threshold, (4) self-moderation is the (achievable) dream, and (5) being part of the moderation team. Table S1 in Multimedia Appendix 4 provides an overview of the themes and illustrative quotations.

#### Theme 1: Moderation as a Pillar of Community Safety

A widely shared opinion among moderators and community members was that moderation is essential: “moderation, it’s just important. It’s got to be there” (community member 6, acknowledger). In particular, moderation was thought to play a critical role in making the community feel safe; a foundational pillar needed for community members to share, interact, and, ultimately, provide peer support. Regardless of how aware members were of the specific moderation policies or day-to-day activities, the simple presence of the moderation team engendered a sense of safety that was considered unique to CommonGround.

It was being overseen or looked after, where, sometimes, on [well-established social media platform], there are some very good support groups, and there are some that are just absolute wild west, and there's people arguing and fighting on them, and they're not healthy places to be. So, there was a sense of safety.

Community member 28, content creator

The fact that the community was for people living with long-term physical health conditions augmented the need for moderation and safety. Community members wished to protect the space from people who did not have long-term conditions, as they felt that they were likely to share sensitive and personal information and experiences related to their health.

[Moderation is] really important, because we're all quite vulnerable. Those of us with chronic illnesses especially, we're all quite vulnerable, and of course, it comes under the whole Equalities Act 2011 and all that, with hate against disabled people being a hate crime, and all that kind of stuff.

Community member 9, content creator

The specific needs of this community also influenced moderator and community members’ opinions on the key areas of moderation. For instance, they shared the view that potentially dangerous topics included the spread of medical misinformation and expressions of emotional crisis or self-harm that required follow-up.

There would be some things where you'd want to be on it really quickly, like if someone's posting about self-harm methods or how they want to end their life. Something like that, you don't want to wait.

Moderator 3

While it was felt that the community was a place to discuss medical and emotional experiences, there was shared concern that these topics require careful review by moderators to ensure that they are discussed in a safe way.

I think for a platform offering peer support, there needs to be some moderation to just make sure that, A, you don't go off down wormholes, that it's not taken over by individuals, the information that's posted is accurate, and that any conflict is dealt with quickly and effectively.

Community member 16, acknowledger

#### Theme 2: The Moderation Blueprint

##### Overview

This theme relates to the policies, community principles, moderator guidance, and training that underpinned the governance of CommonGround. Two subthemes emerged: (1) the importance of this blueprint and (2) the moderator’s experience of translating the blueprint into practice.

##### Subtheme 2a: The Moderation Blueprint Is Essential

In general, community members were satisfied with the moderation and safeguarding policies that were on the platform, recognizing that they were critical for transparently outlining what is and is not acceptable and what to expect in different circumstances. They also provided reassurance that there was a safety net for worst-case scenarios. Typically, community members briefly reviewed the policies at sign-up, often indicating that their review was a relatively light touch and purely to ensure that there were no 'red flags' in the policy. Others made assumptions about the type of content based on their previous experiences or because they considered the owners of the platform (KCL) to be trustworthy.

So, the moderation rules are there, and they should be abided by, and people know if they don’t, they’re parked, and that's how it should be.

Community member 11, content creator

The moderators also felt that the comprehensive policies laid out clear, consistent boundaries for how the platform would be moderated. The moderator handbook, which provided detailed guidance on how to implement the policies, was valued for its specific examples of potential events, as this reduced some of the burden in working out how to respond, including the specific language and tone to use. The moderator handovers were also valuable for keeping up to date on recent activity and highlighting points to review in their moderation shift.

The guidance that we had was quite helpful in that it had quite specific examples for a lot of those scenarios that might come up. And then an example of how you might respond. I found that was quite helpful. And then I guess, as we started doing more of it, you could also see what other people had posted to similar things from the handover document. And then that would, I guess, we'd all respond in a similar way a lot of the time.

Moderator 5

One aspect of the moderation blueprint that split opinions among community members related to the anonymity of CommonGround, with mixed views on whether this constituted a necessary safety feature or stifled members’ ability to establish genuine connections. Other community members felt indifferent, or that the best option would be allow members to select their level of anonymity and give individuals the power to disclose their personal information as they see fit. This call for greater flexibility in anonymity was also recognized by the moderators, who suggested that the moderation blueprint could, and should, be reviewed as the community evolves.

I think maybe going forward is probably about giving people a choice if they want to stay anonymous or not

Community member 44, reader

##### Subtheme 2b: Translating the Moderation Blueprint Into Practice

In general, the moderators agreed that their moderation shifts were quick, manageable, and quite light touch with minimal need to intervene, edit, or delete posts:

It was quite a quick process. Had a checkbox of, check this, check this, check this. And most things had nothing, you know, the flag post, delete posts. Most of them, you’re just checking that there was nothing there.

Moderator 1

The moderators found the guidance in the moderation handbook useful and applicable to the content that came up, and were confident in applying it (Multimedia Appendix 5 provides an illustration of how comments posted by moderators mapped onto advice in the moderation handbook). It was clear that for moderators to successfully do their job, they needed to feel that it was easy to translate the moderation blueprint into the real-world setting of the web-based community. In general, the moderators felt that this translation was easy to do. The main challenge that moderators faced related to the ease and efficiency with which they identified what content was new since the last moderation session. As noted in the methods, during the trial itself, the software developers updated the forum feed layout to display the most recent activity (comment or post) at the top of the feed. The moderation team shared that this technical update helped to reduce the burden of identifying the new content during the trial and that it could be further developed to improve efficiency in a larger, future community. This initial frustration that moderators expressed at the platform-specific issue provided insight into the fact that if their basic need of easy navigation around the community content is unsatisfied, any form of moderation becomes difficult.

Initially, it was more of a system issue. So, we would have to look at comments and threads within the last 24 hours. But initially, the messages were coming up when they were posted, so we wouldn't see the new things immediately. So, having to monitor and try to look at what was new, was really difficult.

Moderator 5

#### Theme 3: The Moderation Threshold

A key experience that moderators spoke about related to navigating the moderation threshold: when to intervene and when to “wait and see” how the situation played out. As time went on, the moderators felt more confident in where the moderation threshold sat, as they learnt how the community worked and reflected on previous moderator activity.

I think when we started to notice that certain moderation styles would actually stifle the engagement from the community, it became quite clear that if you come back to what is the purpose of the platform, that then helps you to make a decision about whether you should do something or not. I guess, like a risk, reward thought process.

Moderator 1

Moderators and community members shared the view that navigating this threshold was particularly difficult around medical information.

[…] By giving space to talk about it, but also disclaiming that that's not a good source, and here's a better source, and having a more balanced opinion, contemplating what they're saying, but also say… It is a fine line, but by acknowledging what they're saying, whilst also posting the counterargument in, it allows people to see.

Community member 6, acknowledger

While the moderators considered their clinical experience useful overall, at times, their experience of dealing with clinical populations and fear of “worst case” scenarios could feel unhelpful. Some spoke of the need to recalibrate their threshold for stepping in to suit the current audience. A couple suggested that including people with lived experience of chronic illness in the moderation team could be beneficial, as they may be better placed to judge when an expression of emotion constitutes emotional distress that requires signposting to one-to-one support.

I wondered sometimes if our professional backgrounds made us more anxious about some of the comments that people made on the platform. As [moderator] said, sometimes you'd get such a small amount of information indicating that somebody is struggling with something. Then because, I guess, my professional background as a clinical psychologist, you see the real peak of distress in terms of the clients that you might work with, particularly when you're working in a physical health context. So then, I suppose, my mind might go to, this person's really distressed. And then you start thinking of all the people that you've worked with clinically, who've been distressed and whether you might need to intervene, and then in terms of managing risk. Whereas actually, it might be that the likelihood is that person's distressed, but it's a really a much lower level of distress than what I might see professionally, that's therefore, less in need of some intervention from the moderators.

Moderator 1

Moderators and community members shared the concern that intervening too early could stifle the community and prevent people from sharing freely. Moderators observed that when they commented, discussion among peers often stopped, leading to a hesitancy to intervene “too early” or unnecessarily. Community members suggested that over-moderation could create a strange dynamic in the community and stagnate conversation. It was also felt to have potential to propel some community members toward “rebellion,” creating further issues.

I think stifling conversations just makes people more likely to try… Well, not even to try and cause trouble, but that's the end result, but it makes them more likely to have extreme views and to go further down the rabbit hole.

Community member 6, acknowledger

Despite the moderators’ anxiety about finding the right threshold, community members often felt that the moderators had struck the right balance. Some suggested that a more authoritarian dynamic may be needed, or necessary, at these earlier stages of community formation to ensure that ground rules are understood and the community grows in a safe manner.

I also felt that there was that kind of safeguarding element with the moderators just being in the background, but they weren't, it wasn't like a very controlling element to it. And they almost showed up as equal participants, almost, but just with that, oh, just a reminder of… It didn't feel like an enforcement group or anything.

Community member 27, content creator

#### Theme 4: Self-Moderation Is the (Achievable) Dream

This theme is related to the shared view that self-moderation was the ideal and the hope that this would evolve in the community over time. Self-moderation would include community members jumping in to moderate potentially problematic conversations and preventing any arguments from emerging. Community members and moderators observed that some members of the community not only regulated what they shared on CommonGround but also intervened in situations where people seemed to be entering potential conflict, share incorrect information, or be in need of emotional support. In interviews, community members commented on their responsibility to think before they post or jump into discussions.

We found after a while, that other users were self-moderating anyway, so we really didn't have to intervene that much, because by the end of the thread, it would have resolved.

Moderator 2

One feature that was built into the platform to support self-moderation was the “flag this post” function that prompted members to determine what they thought was inappropriate and flag it to the attention of the moderators for priority review. This feature was relatively unused by the community, but moderators recognized it as important infrastructure to allow the community to move toward self-moderation if there were to be a larger volume of member-generated content:

[...] One of the functions that was on the platform that wasn't used very much, was users at the platform actually flagging posts that they thought were inappropriate or distressing. That's one way that if it [CommonGround] got bigger and someone wasn't looking at every single post, things would still get identified and then would help to reinforce that ownership of the platform. Because then it's like, what do they [users] find unacceptable? What do they think is going against the principles? […] I think that would be good, maybe to see more of that.

Moderator 5

The opinion that self-moderation would be a superior model of moderation was in part driven by the moderators’ view that hearing from peers is much more powerful than hearing from a moderator.

I felt like the self-moderation was much more powerful in terms of influencing and changing opinion or getting people to think about things in a different way, because it came from a peer.

Moderator 3

However, handing the baton of responsibility over to the community members required careful navigation as moderators balanced their responsibility to keep the community safe and the desire to promote members to have ownership over the community:

[…] The learning from trial and error. Okay, like, this is something that we were concerned about. We were thinking that possibly it could go down that avenue of concern. And actually, what we saw was someone else commented. That ended up fine, we didn't need to do anything. So you learn from the experience, which I think helped us to feel more confident in leaving things be, a bit more, as well.

Moderator 5

Both community members and moderators recognized that a high level of engagement and strong bonds between members were necessary for the community to self-moderate. However, it was recognized that the CommonGround community did not seem to reach this, with more time needed for it to become established and flourish.

I think it's quite a short amount of time to form a community, especially online. And I think maybe there was the start of some of that. At times, certainly. Like what [moderator] was referring to earlier, when there were quite, very supportive responses to people who were clearly distressed and sharing of similar experiences. There were moments where it felt like there was good community support.

Moderator 5

One moderator noted that the inclusion of lived experience experts as part of the moderation team might help to encourage this shift toward self-moderation. When speaking about the future of moderation on the platform, community members noted that the moderation team would likely still be needed in the background. Some community members explicitly identified a continued responsibility of the professional moderators in ensuring platform safety, highlighting that certain scenarios demand their intervention:

Obviously, you’re relying on a moderator to block trolls, or whatever you call them.

Community member 39, never logged in

#### Theme 5: Being Part of the Moderation Team

This theme relates to the positive consequences moderators experienced as a result of being part of a team. The moderators described a sense of shared endeavor in moderating the CommonGround community and had a strong understanding of what their role as a team was, and how their role as a moderator (to keep people safe) was distinct from the engagement team (to promote peer support discussions). Being part of a team helped the moderators to balance the position alongside their other commitments and allow downtime away from the community.

I think we had the right number of people as well. It wasn't that you did it all the time. You did it a couple of times a week at most, which I think was about right. It'd be hard to do it every day, I think.

Moderator 4

The benefit of being part of a moderation team went beyond the practical aspects, allowing for emotional support and encouragement, and opportunities to debrief after tricky situations. One moderator shared that “[…] It felt very open door, in terms of people [the research team and other moderators] being approachable. If we had any questions, it felt very safe and supported” (Moderator 3). In some way, the moderation team’s use of Microsoft Teams chat and handover documents became a form of peer support, with moderators able to seek advice and share how they felt about the role or the community at any time. One moderator recalled feeling well supported by fellow moderators and the research team after they received some negative feedback from a community member whose post they moderated:

[…] Then they wrote another one [post] saying, […] and was a bit shirty. But it was nice because I had a chat with the KCL research team as well, and they said, yes, you did the right thing, which was quite reassuring.

Moderator 4

Being part of a team also helped to build the moderators’ confidence in the role, as they could learn from one another as they implemented moderation policies in a new community. Shared decision-making and reflection helped the team move toward a more efficient method of moderation.

[…] It was quite helpful to have other people dipping in, in between your moderation sessions, because then you get a different perspective on what's already come up. And if there are things that are on that boundary of, is this something we need to do something about or not. Then I found it quite helpful to have someone else's eyes on that. Just my own, I guess, reassurance that we're doing the right thing.

Moderator 1

The range of expertise within the moderator team was also beneficial, particularly when it came to certain topics that moderators were most concerned about, such as diagnosis-related information or medication. Here, it was understood that it was important for the community to be able to speak about it in a safe way.

I do think it was really helpful having some medically trained moderators amongst the group who could be a bit more aware of the comments about medications and treatments and things. Because that was something that I didn't really feel able to moderate that well, because I don't know very much about the medications and the medical treatments for these different health problems.

Moderator 1

### Future Improvements for Moderation

As moderators reflected on their experiences of moderating the CommonGround platform, they highlighted areas where moderation could be improved in the future. Areas for potential improvements and evidencing quotes are presented in Table 3 below.

**Table 3:** Potential areas where the CommonGround moderation could be improved, with corresponding evidencing quotations.

Potential improvement to moderation			Quotes
**General moderation improvements**			
	Include lived experience experts, or clinicians with relevant lived experience, on the moderation team	I think it could have been quite good to have lived experience moderators as well, who would maybe... And maybe that would make a difference to the power dynamic a little bit, in terms of when there were moderator comments, it might be perceived differently? [Moderator 3] I think having people who aren't healthcare professionals, who are more coming from the perspective of that lived experience might help to get a bit more balance there, possibly, in terms of, I guess, the psychological distress part of it. [Moderator 1] If anything, I think getting more community ownership over the platform would be a positive, and maybe having lived experience moderators. But still, the community principles are great, and I think those things are really important. But I think to encourage more community ownership of the platform would help make it a more active platform. [Moderator 2]	
	Explore whether AI^a^ or algorithms could aid moderation	There are lots of massive platforms online that manage to moderate huge amounts of posts. And I guess the way they do that is using algorithms. And they must, because there's no way someone's looking at everything. So, if you scale up, that might be one way to do it. [Moderator 5]	
	Encourage the community towards self-moderation	...One of the functions that was on the platform that wasn't used very much, was users at the platform actually flagging posts that they thought were inappropriate or distressing. That's one way that if it [CommonGround] got bigger and someone wasn't looking at every single post, things would still get identified and then would help to reinforce that ownership of the platform. Because then it's like, what do they [users] find unacceptable? What do they think is going against the principles? […] I think that would be good, maybe to see more of that. [Moderator 4]	
**Platform-specific improvements**			
	Improve the feature(s) for identifying new content on the platform since the last moderation session	I think the efficiency of the moderation could have been way better if you’re flagged to anything new that's come up within 24 hours. […] I think it could be improved by having some way that the platform can flag all of these things are new in the last 24 hours. And then you just go through them, almost like a notification system. [Moderator 1] […] You can almost just have all of them and then you tick them [new comments/posts] off, and you know that they've definitely been moderated. That would be the most efficient way, probably. [Moderator 5] I think with the addition of the notification system, making it super, super clear, what's new, would help it to be moderated. It would help the moderator, if there were more users. I don't feel like it's necessary for the moderator to see everything, because so much of it is just really positive and helpful and supportive, and day to day chats about books and movies and things like that that don't really need a moderator. [Moderator 1]	
	Review the community principle of anonymity	It felt to me that there were a few people who wanted it to be less anonymous. And what “moderator” said earlier, about somebody who wasn't very happy about their personal identifying information being taken out of their posts. I wonder if creating that sense of community for some people is easier, if you are able to share a little bit more information about who you are and what part of the country you live in, and things like that. [Moderator 1]	

^a^AI: artificial intelligence.

## Discussion

### Principal Findings

Online peer support promises a low-cost, easily accessible, and scalable way to support mental well-being and self-management among people living with long-term physical health conditions [1,24]. The risks and potential adverse events that can occur when engaging with peers via web-based communities are well recognized [9,27,31-34]; yet, research into how best to moderate communities to keep them safe is limited, particularly in the context of new web-based health communities [37]. In this work, we explored the perspectives and experiences of both community members and moderators together to evaluate the acceptability and feasibility of our co-designed moderation procedures and identify areas for future improvements. Overall, community members and moderators both had positive views of our moderation procedures. Moderators found it feasible to implement the polices but identified opportunities to improve moderation as the community culture evolves. Based on these findings, we provide recommendations to guide the development and implementation of moderation practices for new communities (Table 4).

**Table 4:** Recommendations on how to moderate web-based peer support communities.

Recommendation	Description
1. Coproduce clear moderation policies that reflect best practice	Develop moderation in collaboration with people who have lived experience to ensure alignment with the values of the target end user. Clearly define acceptable and unacceptable behaviors and identify key topics of moderation concern that should be prioritized. Be transparent in why, when, and how any moderation will happen. Lived experience experts should define what is acceptable and appropriate around topics that are important to them, such as crisis support and anonymity. The tone and language that will be used should be agreed upon enhance the acceptability of moderation practices.
2. Establish clear escalation and referral pathways	Clearly define what support is available for community members in crisis. Ensure moderators are trained in handling high-risk situations in accordance with the escalation and referral pathways. Consider the pathways to be taken when handling complex or ambiguous cases.
3. Determine how the moderators “speak” and be consistent	Tone and language can dramatically affect how moderation is received in the community, with inappropriate tone or language stifling conversation or creating a “big brother” culture. Consistency is key for moderation to perceived as fair and reduces the risk of members developing a specific attachment to individual moderators. Sharing template phrases and responses to common situations can help reduce moderation burden. In health-related settings, tone and language is particularly important in relation to moderating medical topics and emotional distress.
4. Communicate moderation and moderation decisions transparently	Share moderation policies transparently in easily accessible formats. Provide clear and respectful feedback to members who are moderated, referring to the relevant sections of the policies. This helps members understand boundaries, reduce hostility toward moderators and minimize the likelihood of future moderation issues.
5. Create features that encourage self-moderation among the community	Having a community that keeps itself safe and moderates itself (eg, encouraging peers to seek support from their general practice doctor if medical advice is being discussed) with minimal intervention from the moderation team is the ideal. When creating the interface of the online peer community, co-create features that encourage self-moderation, such as “flag this post”.
6. Be responsive and evolve moderation processes as the community evolves	Moderation should evolve with the community. Regularly review what is working well, what is burdensome, and how moderation is impacting members and moderators. Early on, there is a greater need to establish clear boundaries. Over time, community members will grow ownership over the community, and moderator behavior should shift to nurture and accommodate self-moderation. Adjust the features of the interface and moderation practices as needed. Ensure to seek the perspectives of both community members and moderators to ensure a comprehensive understanding of what is working well and what needs adapting. For example, the moderation team told us that they found it challenging to track new content since their last session. In response, we improved the visibility of new content with new timestamps and post ordering in the community forum. Guidance on gathering feedback: Moderators are encouraged to reflect on how different moderation approaches and practices are being perceived by the community by reflecting on the community engagement/comments that follow any intervention by moderators. By considering what was done and how the community reacted to the moderation behavior, moderators can adapt their approach to improve how it is delivered and received. Additionally, by reflecting on commonalities among posts that users “flag” to the attention for the moderations, key areas that need further development or greater moderation attention can be detected. With the emergence of AI^a^ and algorithms that can monitor community activity, in the future, there could be scope to explore how AI could assist moderation and help identify where moderation is working well or needs adjusting. Whilst further work is needed to explore the usefulness and appropriateness of AI-assisted moderation, this emerging area could offer exciting opportunities for streamlining and facilitating community moderation.
7. Ensure moderators feel supported	Have ways for the moderators to connect outside of the peer community, for instance, through a Microsoft Teams chat. Creating a space for moderators to discuss situations and come to a collective decision, decompress from emotionally-taxing situations, and share learnings or updates can be beneficial. Provide regular training specifically related to the community’s policies, particularly when these change over time.

^a^AI: artificial intelligence.

To our knowledge, this is the first study to integrate the experiences of community members and moderators to understand moderation in a newly launched peer support community. It was universally agreed that moderation is a critical pillar of community safety, reflecting the perspectives of members [31] and moderators [9] of other established communities that moderation is essential for creating a safe space and mitigating risks. As in other web-based health communities, we also found that the community members and moderators had particular concerns about the need to moderate medical misinformation [9,31,37]. This highlights the importance of tailoring moderation practice to the unique aims and sensitivities of the specific community, and the need to identify these through collaboration with the target audience (Recommendation 1, Table 3). While moderators reported a few instances where they were required to enforce the moderation policies, they felt that the moderation blueprint with the clear escalation procedures was appropriate and applicable to the community. The minimal need for moderator intervention may reflect the relatively low activity levels on CommonGround, where fewer user-generated posts naturally reduced the need for moderation. Equally, it may be because members understood and were happy to abide by the moderation policies [30]. Community members found “no red flags” in the moderation policies, suggesting that these were aligned with their expectations and moderation needs.

The apparent success of moderation also suggests that co-designing moderation practices is critical in ensuring their acceptability among the community, and that having clear boundaries and explicit guidance for moderators can lay the foundations for successful moderation from “Day One” of the community (Recommendations 1-3, Table 4). Community members valued being able to easily access the moderation policies when needed, underlining the need to share moderation practices transparently, which can enhance legitimacy, perceived consistency, and accountability of moderation ([51]; Recommendation 4, Table 4). Improving the communication of reactive moderation decisions may help to increase understanding, promote adherence to norms, and improve future behavior [51,52]. Together, these findings underscore the importance of having clear, comprehensive moderation practices tailored to the community, including the specific content of policies and the transparency, tone, and language of communications. Where the capacity to codesign moderation policies and practices is limited, examples from existing communities can be drawn upon, particularly as key moderation practices and experiences are likely common to any online peer community, such as the use of peer moderators [30].

By integrating the perspectives of community members and moderators, we gained unique insight into areas of moderation that may require review and adaptation as the newly formed CommonGround community evolves. The anonymous nature of web-based communities is often seen as a double-edged sword: anonymity can facilitate stigma-free and honest posting for some [53], whilst others feel that anonymity hinders the ability to form the meaningful and enduring social connections that are the cornerstone of peer support [54,55]. We found similar mixed opinions among our community members. The desire for reduced anonymity may grow as community members become more willing to share personal information if it makes accessing social support from peers easier, even if this could introduce other risks [56,57]. The reputable KCL ownership and branding positively influenced perceptions of safety. However, as some members appreciated the anonymity or acknowledged its value for others, offering members the ability to choose their preferred level of anonymity may be appropriate. Future work should explore with lived experience experts how to implement features that allow personal control over anonymity to ensure that anonymity can serve as a facilitator to engagement for those who desire it, while also removing this potential barrier to social connection for others [58,59]. These divergent views on a core aspect of CommonGround’s moderation practice highlight the importance of reflecting on moderation practices by listening to the community and adapting as necessary (Recommendation 6, Table 4).

The moderation practice and style needed for CommonGround as a new community were recognized to be different from those of larger, well-established communities. In well-established communities, community members can be seen to be actively self-moderating by detecting medical misinformation, questioning information shared, reminding peers not to give medical advice, and reporting trolls to moderators [31,37,60]. While CommonGround moderators and community members agreed that peer-to-peer moderation could be more powerful and feel less punitive, they recognized that CommonGround could not self-moderate yet. The limited volume of user-generated content and the weak sense of community and ownership among members is likely to have hindered the emergence of self-moderation. The shift toward self-moderation must occur organically, as the premature withdrawal of moderators could harm community cohesion and reduce perceptions of safety if members are not comfortable acquiring moderation responsibilities [31]. Indeed, as prior work often cites “super users” (ie, those who engage with the community often, and are often observed to be generating larger volumes of content than the average user) in contributing to how this culture evolves, who often only emerge as a community evolves and may also facilitate the shift toward self-moderation [61]. As voiced in our research and prior work [31], there may always be a need for professional moderators to deal with high-risk or serious issues, even if the community takes ownership over other moderation activities. Although used infrequently on CommonGround, the moderators valued features like “flag this post” as critical tools for encouraging a community towards self-moderation. Indeed, moderators of other web-based health communities have urged members to use such self-moderation tools more to help shape moderation norms by defining what content they deem unacceptable [30]. Therefore, while the community culture needed for self-moderation cannot be artificially created, the features and functions that support self-moderation can be implemented early (Recommendation 5, Table 4). Future work should evaluate member and moderator perspectives as a community evolves through different stages of self-moderation to determine what additional practices may help support a successful transition.

Few studies have explored the experiences of moderators, but some key challenges consistently emerge [37]. First, a common challenge lies in efficiently identifying new content to reduce the risk of unmoderated posts escalating into worst-case scenarios [9,37]. Moderators were also mindful of how this challenge could intensify as the community grows, underscoring the need to develop and test novel tools that enhance moderation efficiency as CommonGround is scaled. Another challenge that our moderators faced was navigating the moderation threshold where they must evaluate whether to take action [9,30,51]. The views of the moderators echo those of moderators in other communities, who want to prioritize safety but are concerned that premature or overbearing moderation can stifle conversation and potentially damage community culture [62]. Moderators observed that once they commented, the community ceased to comment further. This pattern has been observed in other communities [62], with moderators interpreting this to reflect disengagement from the community. However, members typically felt that the moderators had struck the right balance, suggesting that the quiet comment section may instead reflect the generally low engagement we had on CommonGround, where most members were “readers” preferring to observe posts rather than actively create content. This disconnect between how moderators believe their actions are perceived and how the community actually experiences them highlights the importance of considering both perspectives, particularly if informing changes to moderation practices (Recommendation 6, Table 4).

The benefits that the CommonGround moderators experienced as a result of operating as a team have been documented among other moderation teams. For instance, the CommonGround moderators shared how it became easier to navigate the moderation threshold over time and as a result of seeking support from their team, as they were able to exchange experiences to align their moderation practice, seek emotional support, and access the expertise of other members for second opinions on complex situations [63]. This adds to the literature highlighting how the moderation team becomes a form of peer support in itself that can help improve moderation efficiency, consistency, and reduce the moderation burden [9,64]. This highlights how it is important to consider how best to support moderators whilst they are supporting the community and give them a dedicated space to connect and support one another (Recommendation 7, Table 4).

### Limitations

There are some limitations that should be considered for the interpretation and transferability of our findings and recommendations. First, this study explored the perspectives of community members and moderators who participated in the new community that was live for only 3 months. Many community members shared that they did not engage with CommonGround as they would have liked to due to personal circumstances or having limited time available during the 3-month trial. While lower levels of engagement and forum activity could be expected during early stages of community development, the experiences shared in this work are contextualized within a small community with relatively low engagement and sense of community in a research setting. As such, our findings and recommendations may not apply to communities launched with larger populations or with high volumes of user-generated content from the outset. Second, our findings should be interpreted relative to the nature of our moderators: professional, paid moderators with clinical expertise who were trained specifically about the moderation practices for CommonGround. Prior research suggests that professional moderators are often perceived as more trustworthy and credible than informal moderators, particularly around their moderation of medical topics [31]. Indeed, our moderators were aware of how their clinical expertise influenced their approach to moderation, and therefore, our findings may not generalize to moderation teams of volunteers, peers, or those without clinical or moderation backgrounds. Third, there is a potential for selection bias in the community members’ interviews, as those who consented to participate may have more positive views of web-based peer forums or the moderation and governance of CommonGround. However, we did purposefully sample community members based on their engagement levels, probing those who had never logged in or only logged in a few times for their underlying reasons, motivations for low (or no) engagement, to unpack whether moderation or governance played a role in their disengagement. Fourth, although community members were anonymous to one another, they were aware that the moderators could link their pseudonyms to their personal details if required for moderation purposes. This awareness that they could be identified in exceptional circumstances may have influenced what members posted, meaning that the CommonGround environment may not directly reflect non-research anonymous communities.

All moderators and community members needed internet access to participate in the web-based community and interviews or focus groups. While 96% of households in the United Kingdom have internet access [65], for those without or who have limited access, their ability to engage with the community and interviews may have been limited. Additionally, some participants may have experienced barriers in accessing CommonGround privately, which may have had a downstream impact on the content of the community forum, and therefore the moderation needed [66]. Future work should consider how digital exclusion and privacy influence the nature of web-based communities, in particular their moderation.

### Conclusion

Our work adds to our understanding of the critical role moderation has in shaping safety in online peer support communities designed to support mental well-being, and the importance of creating appropriate and trustworthy governance that is specifically tailored to the needs of the community members. By exploring perspectives on moderation in a new community and by integrating the experiences of both community members and moderators, we have gained unique insights into how to enhance our codesigned moderation practices going forward. We have provided a transparent account of the creation, implementation, and reflection on our moderation policies and governance, and recommendations on how to approach the moderation of new communities in the future.

## Appendix

Multimedia Appendix 1 SRQR checklist.

Multimedia Appendix 2 Screenshots of the final prototype of CommonGround.

Multimedia Appendix 3 Interview and focus group topic guides.

Multimedia Appendix 4 Theme descriptions and further evidencing quotes.

Multimedia Appendix 5 Comparison of moderator comments on CommonGround to moderation guidance.

## Abbreviations

EQUATOR: Enhancing the Quality and Transparency of Health Research
fRCT: feasibility randomized controlled trial
KCL: King’s College London
SRQR: Standards for Reporting Qualitative Research
