# Supplementary material for: Insights and Recommendations From Moderators and Community Members for Keeping Online Peer Support Safe: Thematic Analysis
Source: J Med Internet Res. 2026 Mar 12;28:e81943. doi: 10.2196/81943 (PMC13022541; doi:10.2196/81943)

## Appendix 2

**Screenshots of the final prototype of CommonGround**

Screenshot S1. The landing page of CommonGround


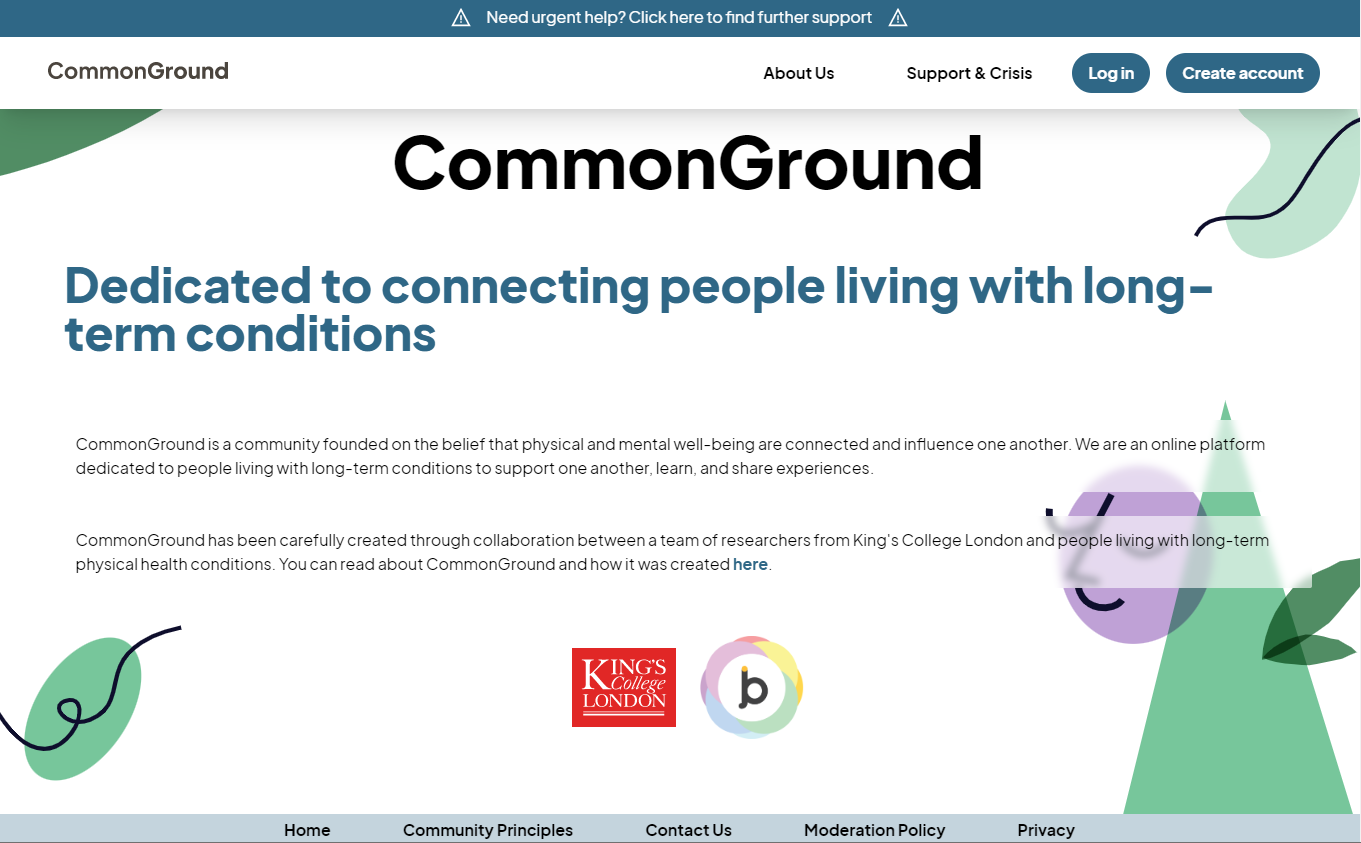


Screenshot S2. The community feed of CommonGround (peer support component)


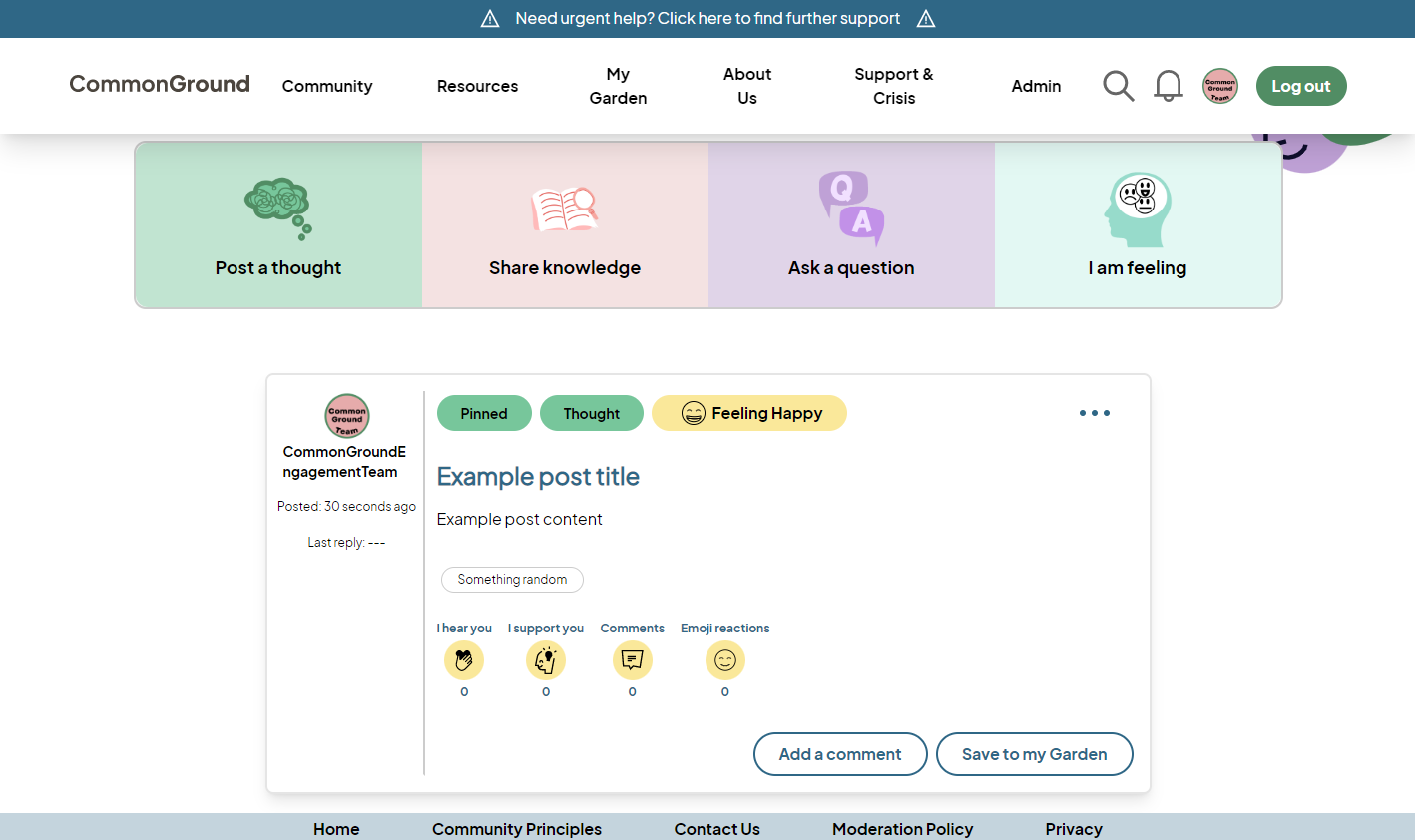


Screenshot S3. The resources page of CommonGround (psychoeducation component)


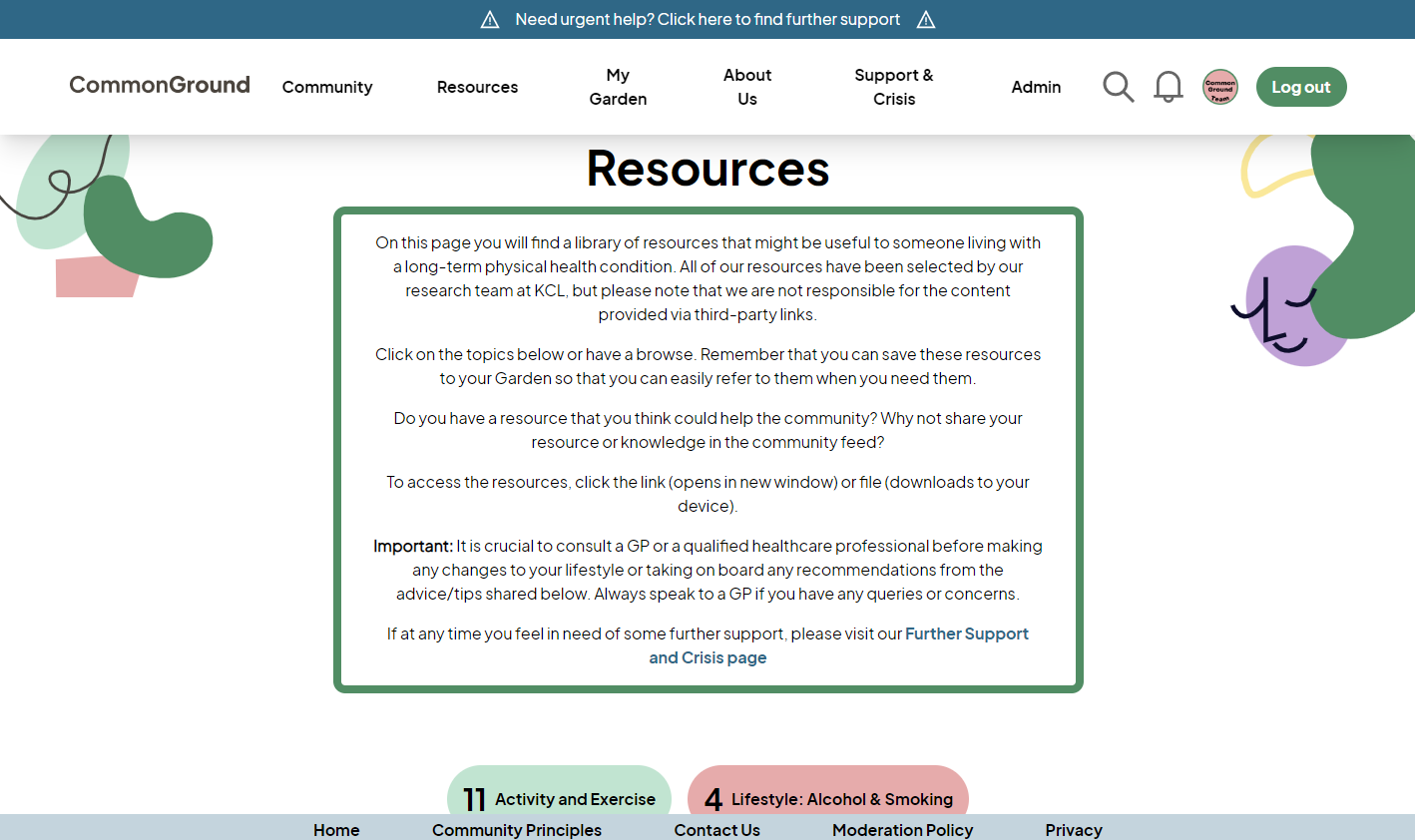


Screenshot S4. The ‘My Garden’ page of CommonGround


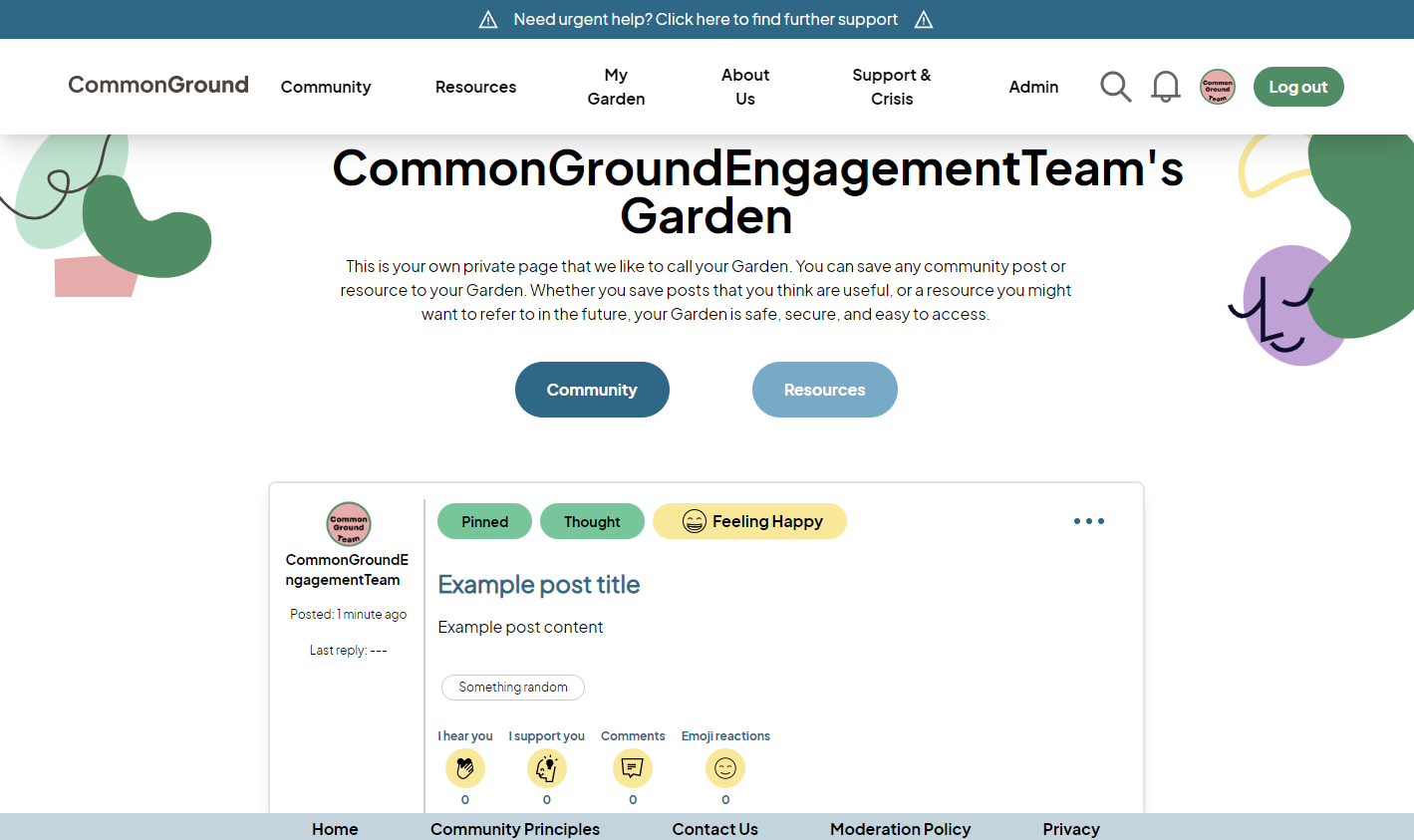


Screenshot S5. Search box and search results page of CommonGround


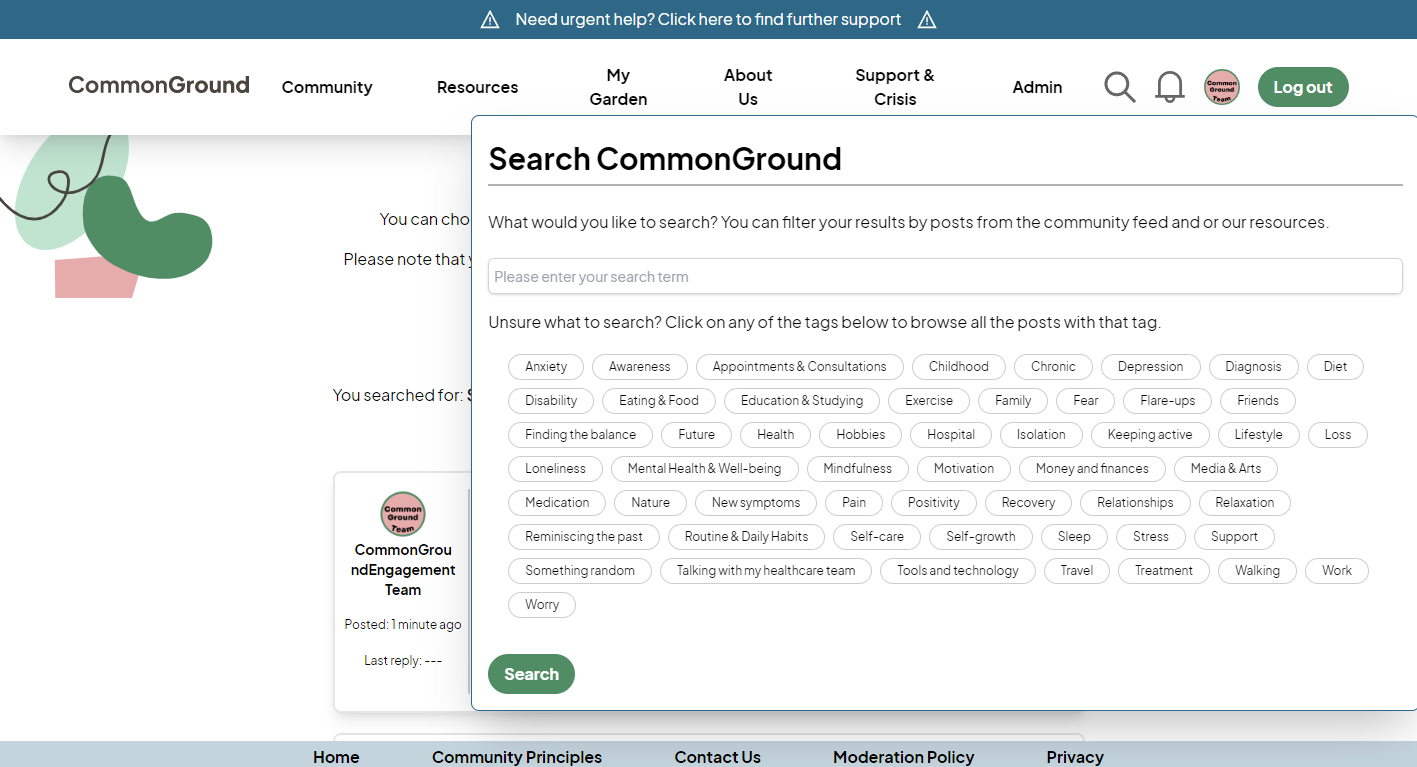


Screenshot S6. Further support and crisis page


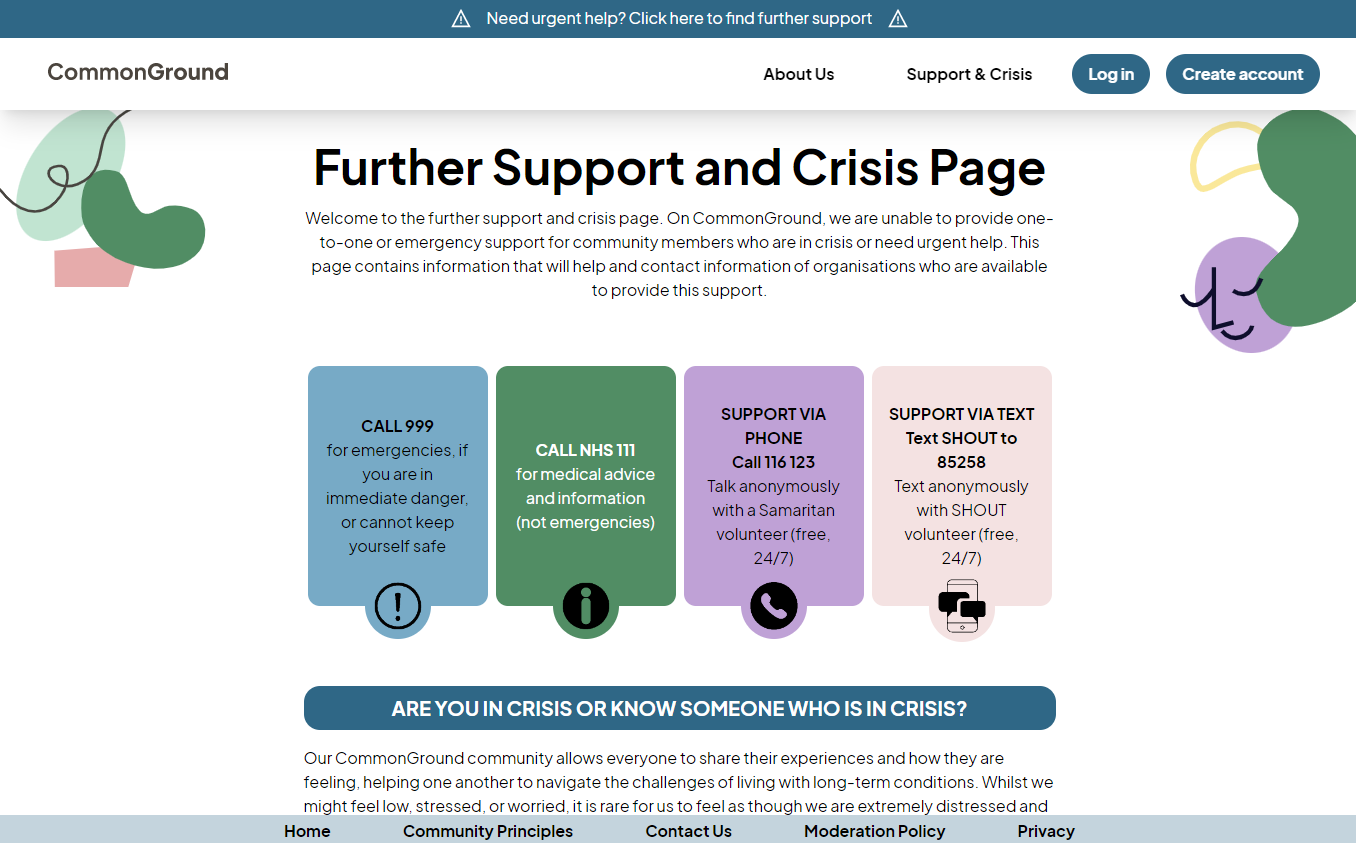


Screenshot S7. Moderation policy page


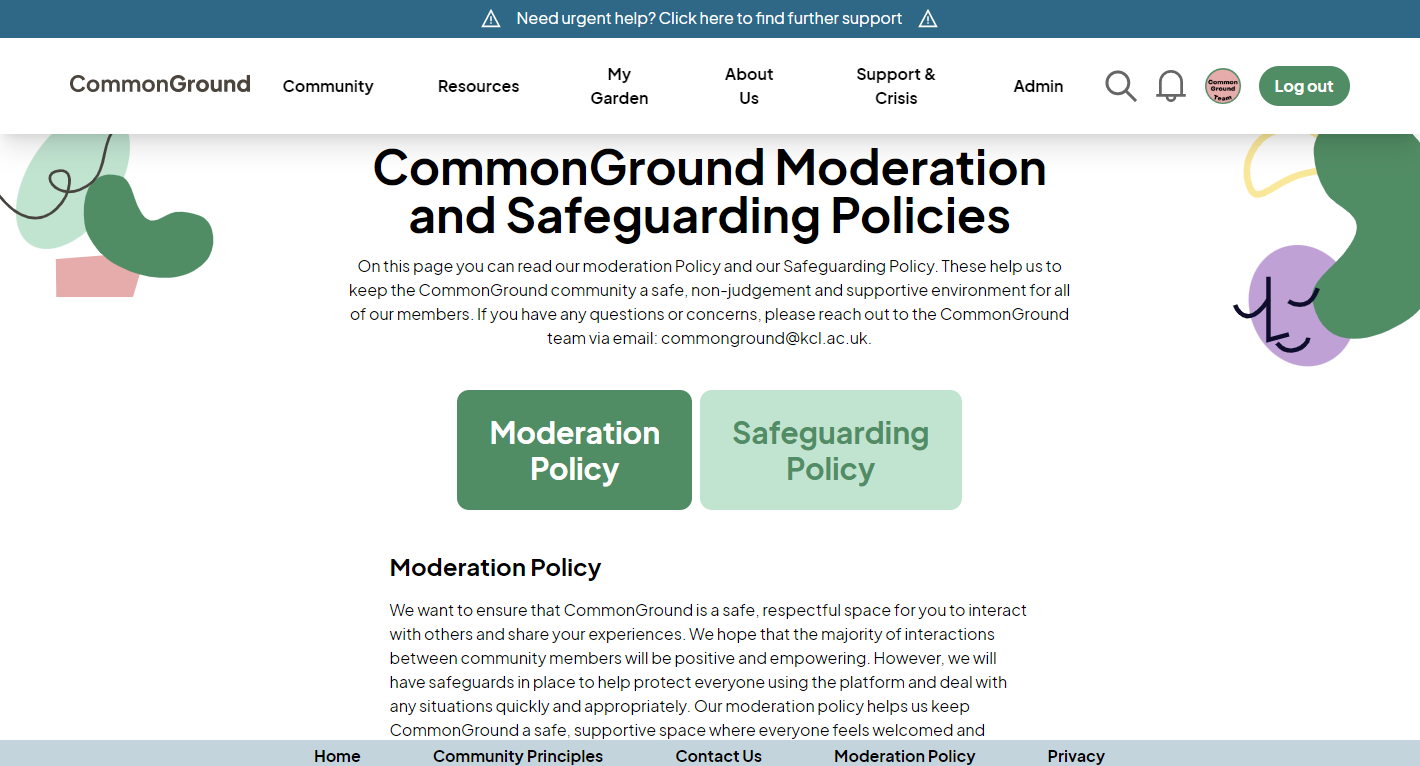


Screenshot S8. Admin panel


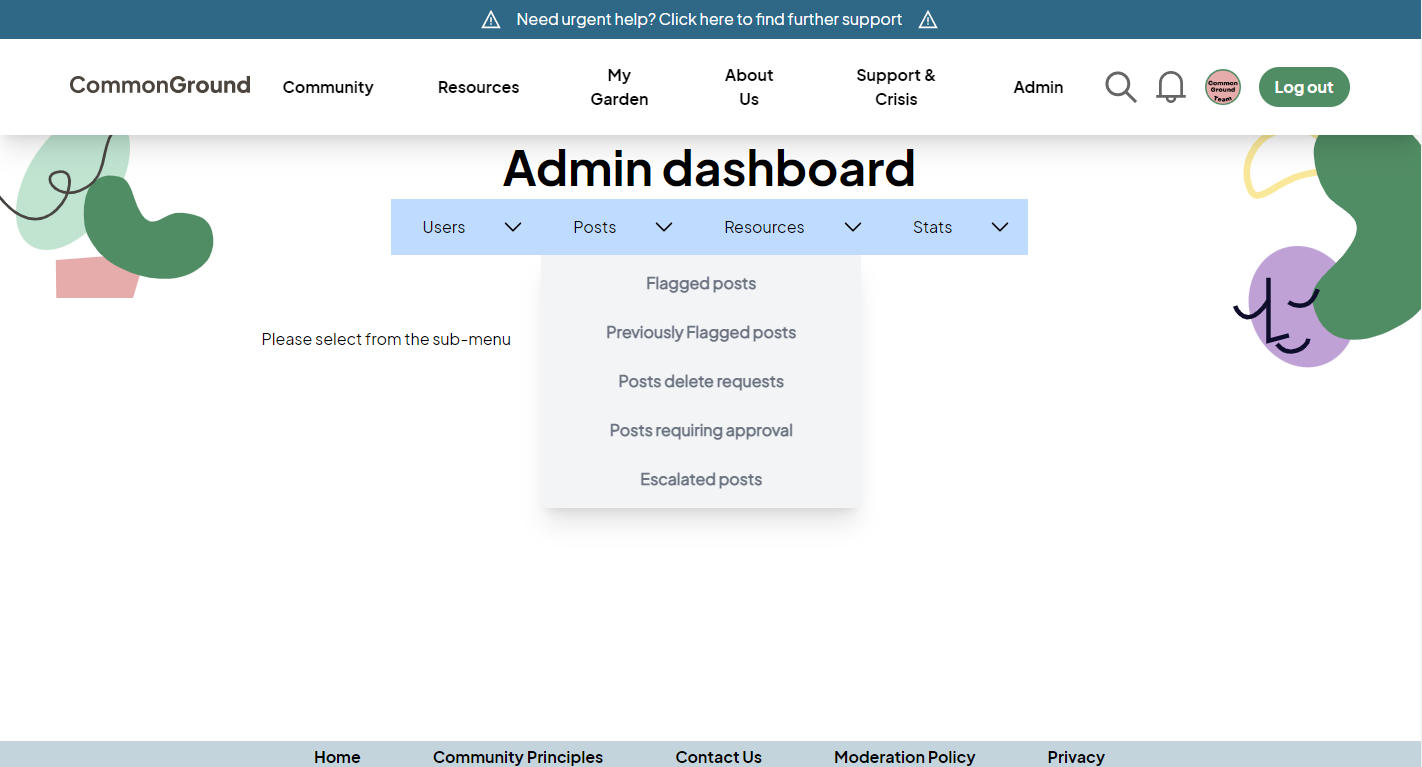


Screenshot S9. Admin panel: Delete posts that users have requested to have deleted


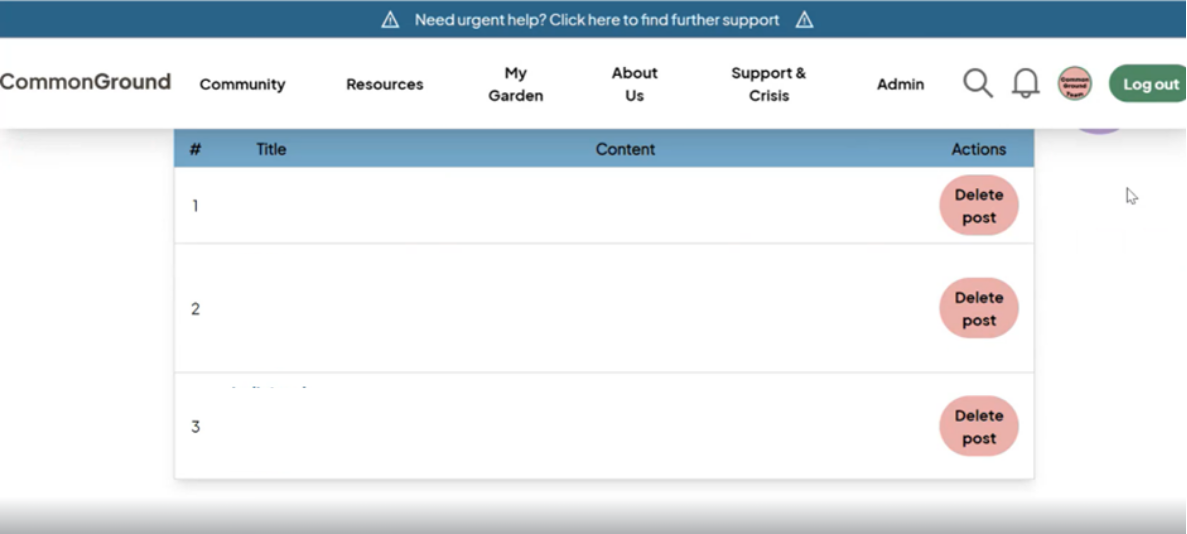


Screenshot S10. Admin view: Moderation notifications


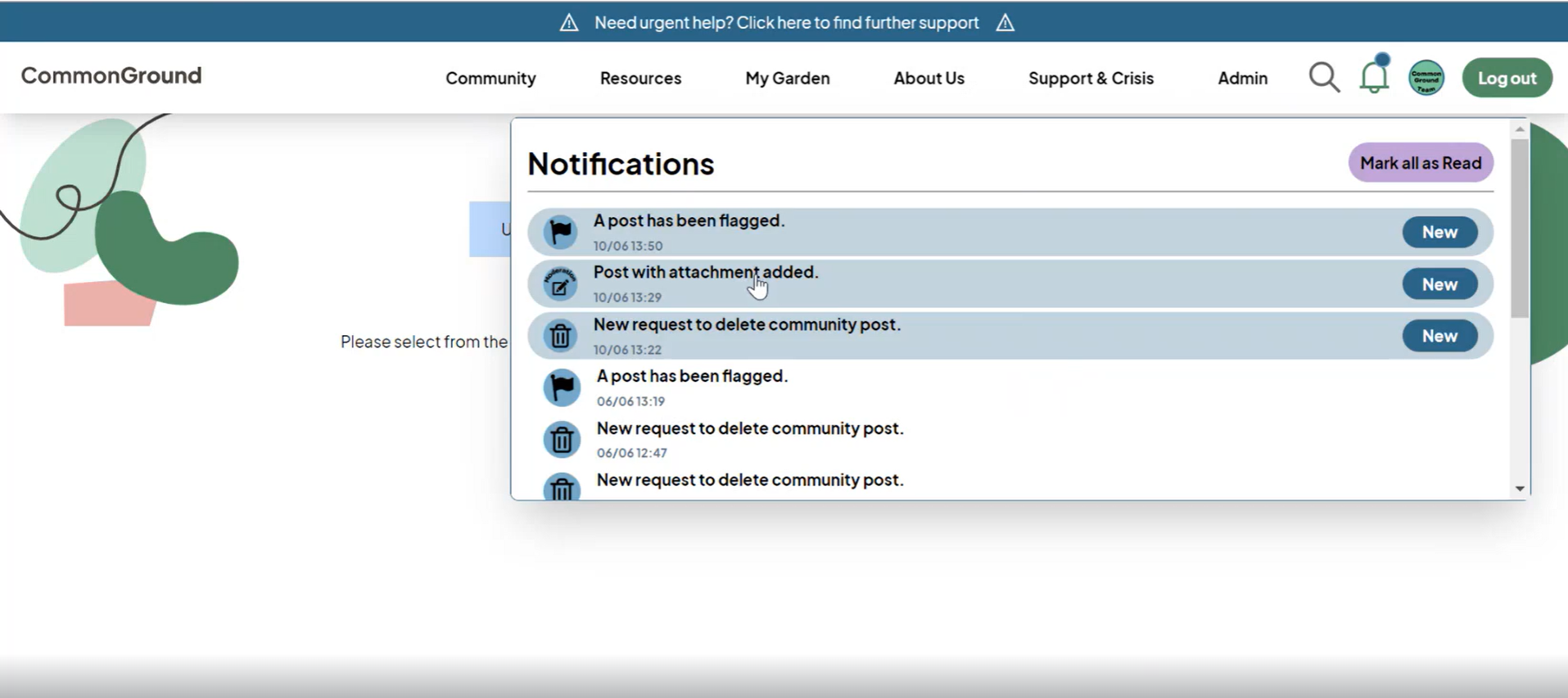


Screenshot S11. Admin view: Reviewing a post with an attachment before it is ‘live’ in the community forum.


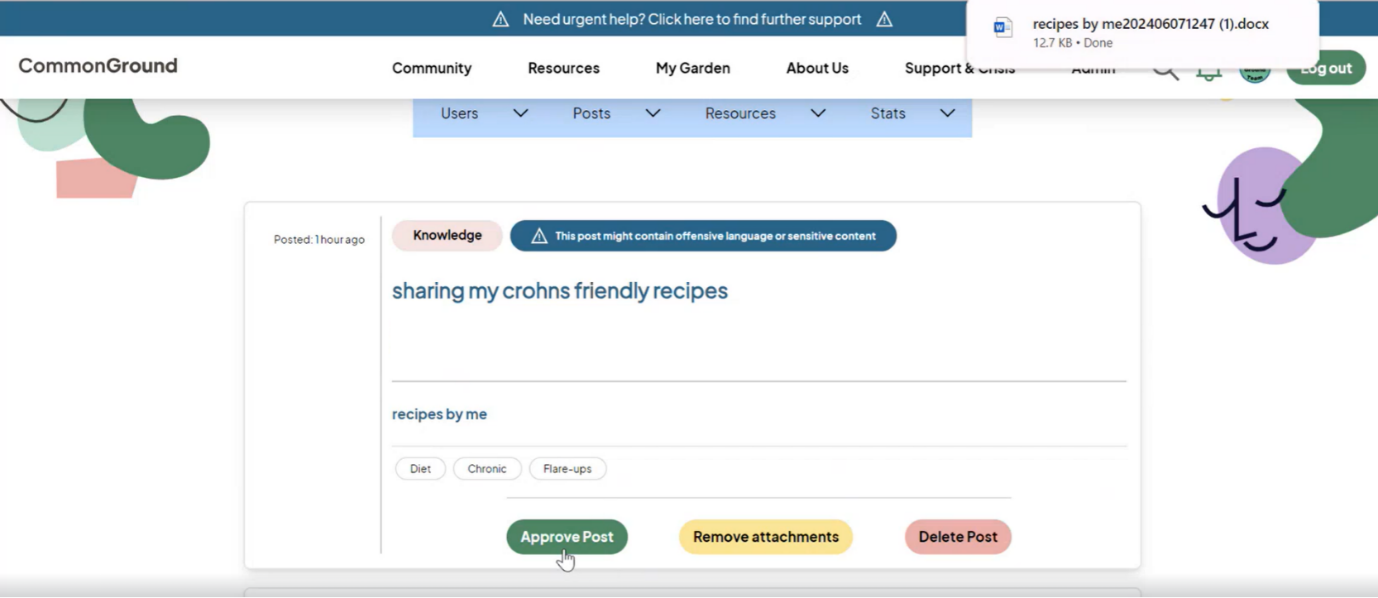


Screenshot S12. Pop-up window for community members to flag a post to the attention of the moderators.


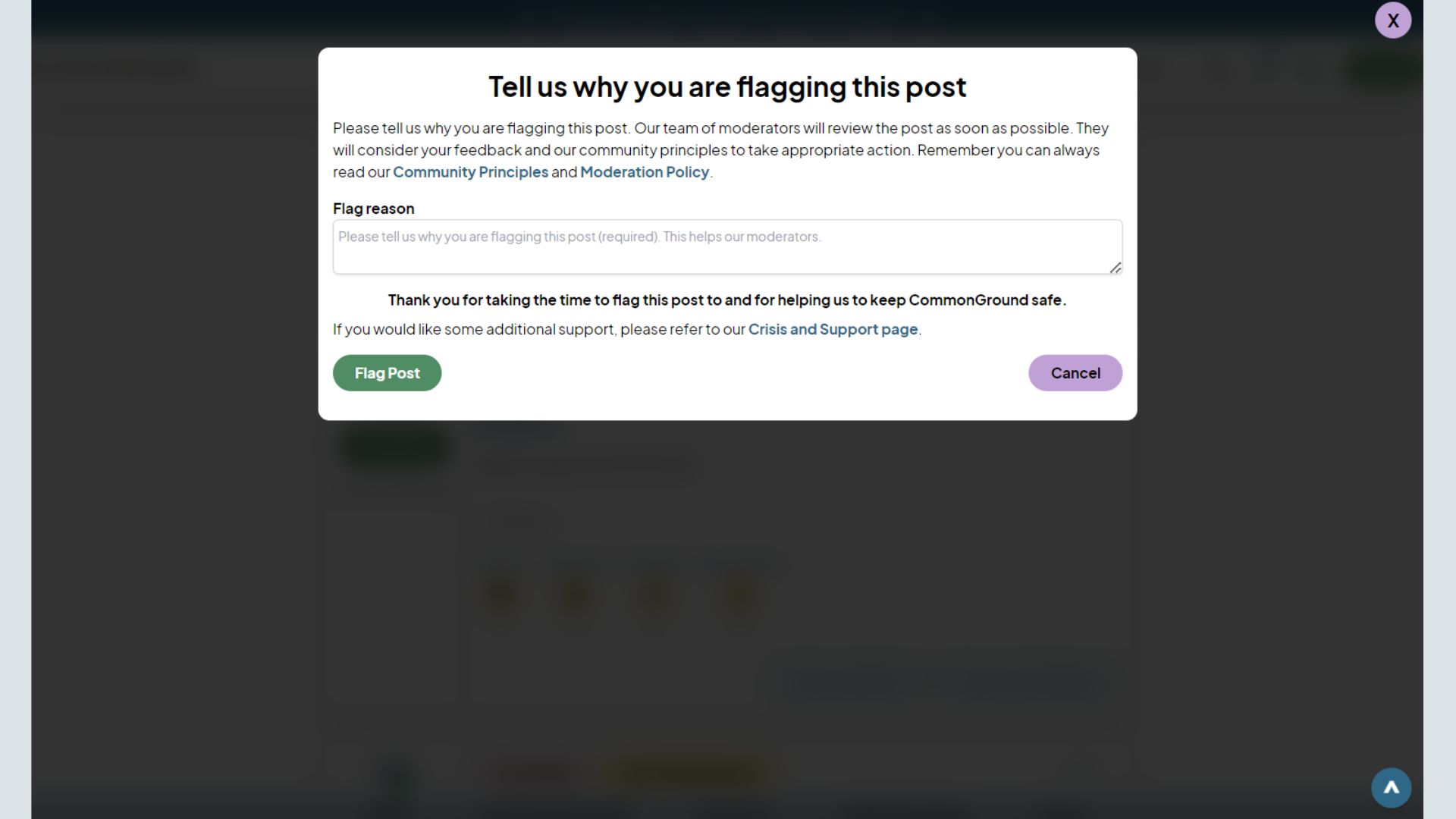


Screenshot S13. Privacy toggles on community members profiles.


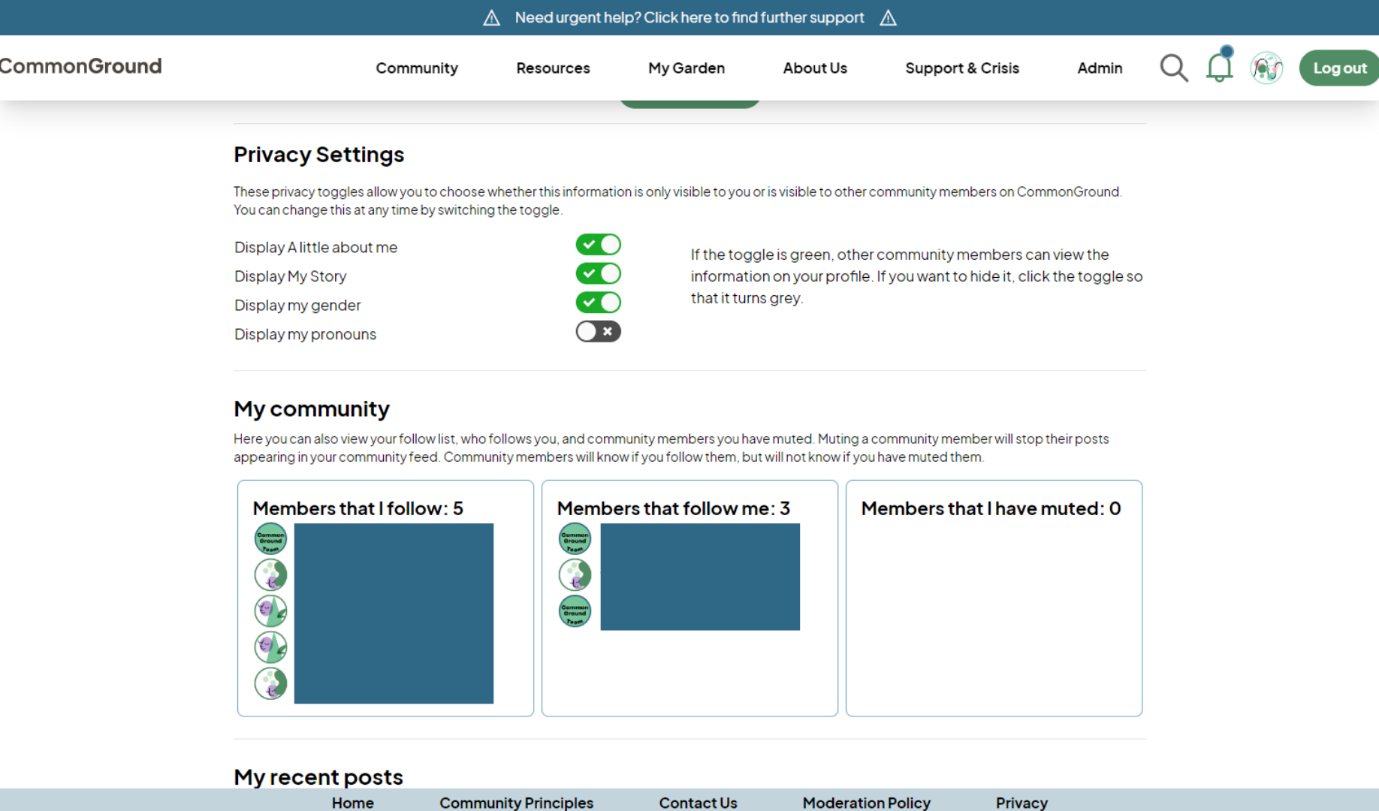

Supplement: Multimedia Appendix 2 [file jmir_v28i1e81943_app2.docx]
