# Supplementary material for: Insights and Recommendations From Moderators and Community Members for Keeping Online Peer Support Safe: Thematic Analysis
Source: J Med Internet Res. 2026 Mar 12;28:e81943. doi: 10.2196/81943 (PMC13022541; doi:10.2196/81943)
Supplement: Multimedia Appendix 3 [file jmir_v28i1e81943_app3.docx]

# Appendix 3: Interview and focus group topic guides.

## [1] Community members topic guide: Never signed up or never logged in.

*Introduction*

1. Why were you first interested in the Common Ground project?
2. Where do you usually go for information on managing your mental and physical health?
3. What does peer support mean to you? Is peer support something you have come across before?
4. Did you ever log into Common Ground? Did you use the platform?

*Engagement with CommonGround*

1. What would you say the main reason(s) for not using CommonGround were?
2. CommonGround included a library of evidence-based self-help information to help people with long-term conditions manage their physical and mental well-being. What do you think about these psycho-educational resources on CommonGround?
3. The peer support platform included an online discussion forum called the community page. You could create posts, ask questions, share your experiences, react and comment on other people’s posts. What do you think about this feature?
   - If you did not use it, why not?
4. The My Garden space was a section where you could save things to a personal library. What do you think about this feature?
   - If you did not use it why not?
5. The moderation team role was to make sure that people followed the Community Principles when using CommonGround. Their role involved reviewing, editing and deleting posts and guiding discussions were necessary.
   - What were your experiences (if any) of the Moderation team during the trial?
6. The Engagement Team role was to encourage engagement through weekly emails and through posts, summarise trending discussions and shared relevant topics for people living with LTCs.
   - What were your experiences (if any) of the Engagement Team during the trial (on the platform and via email)?

*Features and Functions of CommonGround*

1. If you did create a platform, how did you find creating an account on the platform?
2. What did you think about CommonGround being an anonymous platform?

*Trial Features*

1. How did you find the ‘wait period’? As a reminder, the wait period happened after you told us you were interested, before we were ready to launch the trial and got you to complete your first questionnaire.
2. How did you find the length of the study overall? How did you find the length of the intervention period (i.e., the three months of CommonGround access)?
3. How did you find the questionnaires that we asked you to complete?
4. Throughout the research trial, was there anything that you found difficult or did not understand?
5. How did you feel about CommonGround being a place for people with any physical health condition?

*Future of CommonGround and Closing*

1. Thinking about the future, what other features/functions would you like to see added to a platform such as CommonGround?
2. Is there anything you think could have been done differently in our study and what we asked you to do?
3. If CommonGround was freely available in the future, would you use it? Why or why not?
4. Who do you think would benefit most from CommonGround? Who do you think that CommonGround is designed for?
5. Reflecting on everything we have spoken about today; do you think being involved in the study helped you in any way? How?
6. Overall, if you were to describe your experiences of participating in this study in 3 words what would they be?

*[end]*

## [2] Community members topic guide: Platform users.

*Introduction*

1. Why were you first interested in the Common Ground project?
2. Where do you usually go for information on managing your mental and physical health? Why do you choose these sources of information?
3. What does peer support mean to you? Is peer support something you have come across before?
4. Did you ever log into Common Ground? Did you use the platform?

*Platform use*

1. What did you use CommonGround for? What motivated you to login?
2. The peer support platform included an online discussion forum called the community page. You could create posts, ask questions, share your experiences, react and comment on other people’s posts. What do you think about this feature? Did you use it?

**If not,** why not?

- Prompts: Was it related to the topics and content? Was there something else you would like to see covered?
- Was it related to how accessible or inaccessible the resources were? Your time? The reasons why you joined the platform?

**If you did:** What parts of this feature did you like? How did you use the community page?

1. CommonGround included a library of evidence-based self-help information to help people with long-term conditions manage their physical and mental well-being. What do you think about these resources on CommonGround? Did you access them?

- If did not use, why did you choose not to look at the resources on CommonGround?
- If you did: How did you use the resources and how did you decide what resources to look at?

1. The My Garden space was a section where you could save things to a personal library. What do you think about this feature?

- If not, why not?
- If you did: how and why did you use it?

1. When and how did you use CommonGround?
2. What did you like about using CommonGround?
3. Was there anything you did not like about using CommonGround?

*Features and Functions*

1. How did you find creating an account on the platform?
2. What did you think about CommonGround being an anonymous platform?
3. What were your experiences (if any) of the Moderation Team on CommonGround? What effect did they have on CommonGround? Tell us about any noticeable interactions you had or observed.
4. What were your experiences (if any) of the Engagement Team during the trial (on the platform and via email)?
5. Could you tell the difference between the engagement and moderation Team?

*Peer support and suitability*

1. Did you feel that a community was formed on CommonGround?
2. How did you feel about CommonGround being a place for people with any Long-term health condition?

**Trial Features**

1. How did you find the questionnaires that we asked you to complete?
2. Throughout the research trial, was there anything that you found difficult or did not understand?
3. How did you find the length of the study overall? How did you find the length of the intervention period (i.e., the three months of CommonGround access)?
4. How did you find the ‘wait period’? As a reminder, the wait period happened after you told us you were interested, before we were ready to launch the trial and got you to complete your first questionnaire.

*Future of CommonGround and Closing*

1. Thinking about the future, what other features/functions would you like to see added to a platform such as CommonGround?
2. Who do you think would benefit most from CommonGround? Who do you think that CommonGround is designed for?
3. If CommonGround was freely available in the future, would you use it? Why or why not?
4. Reflecting on everything we have spoken about today; do you think accessing CommonGround helped you in any way? How?
5. Overall, if you were to describe your experiences of participating in this study in 3 words what would they be?

*[end]*

## [3] Moderator focus group topic guide

*Moderators Experience*

1. Why were you first interested in the CommonGround project? Why did you decide to take part as a moderator?
2. What does peer support mean to you?
   - What are your current views of peer support?

*Functions and Features*

1. How easy or hard is it to navigate the platform as a moderator?
2. What were the technical issues or limitations with the platform (if any)? How often do technical problems (e.g., crashes, bugs) impact your ability to moderate?

*Content Management*

1. What type of moderation did you do the most? I*f mainly editing/deleted posts / comment:* What were the most common topics/discussions that required moderation? What actions did you do least often/never did?
2. Most often: How they found the process. Did they feel they had effective tools (i.e., features and functions of the platform)? Effective guidance on moderation action? Effective support? Effective Training?
3. What were the biggest challenges of moderation?
4. What worked well during moderation?
5. When thinking about moderation training, moderation policies and principles, what was the reality of implementing them? How did the expectations compare to the reality of moderation?

*Security and Privacy*

1. If any, what safety/privacy/security concerns did you have during moderation?
2. Did you experience safeguarding concerns?

*Moderation sessions/workload*

1. Time Management: How much time does moderation require daily? Is flexibility needed or did you find the set time doable?
2. Did you find that each session had enough time?
   - Do you think the platform not being moderated 24/7 worked? If so/if not, why?
   - Do you think weekend moderation is necessary? Why/why not?
   - What other considerations might be necessary if the community was larger?
3. Experience of the process of sharing moderation/communication:
   - What was your experience of the handover process?
   - How did you find the communication and collaboration between moderators? Do you think the system for communication and collaboration between moderators could be improved for future platform moderation?

*Future of CommonGround*

1. What changes or improvements (if any) would make moderation more manageable and sustainable in the longer term?

*Perception of Users experience*

1. How do you perceive users' experience of the platform? What did user’s appear to use the platform for?
2. How did user’s interact with the moderation Team? How do you think the moderation Team influenced people's experience of the platform? Positively or negatively?
3. How did user’s interact with the Engagement Team? How do you think the engagement Team influenced people's experience of the platform? Positively or negatively?

*Community and suitability*

1. Did you feel that a community was formed on Common Ground? What things contributed to feeling like there was / was not a community? If not how could things be improved in the future?
2. How did you feel about CommonGround being a place for people with any physical health condition diagnosis?
3. If the platform was to be offered in the future are there any key changes you would make to the platform?

*[end]*
