# Supplementary material for: Insights and Recommendations From Moderators and Community Members for Keeping Online Peer Support Safe: Thematic Analysis
Source: J Med Internet Res. 2026 Mar 12;28:e81943. doi: 10.2196/81943 (PMC13022541; doi:10.2196/81943)
Supplement: Multimedia Appendix 4 [file jmir_v28i1e81943_app4.docx]

# Appendix 4.

# Table S1. Theme descriptions and further evidencing quotes.

| **Theme and theme description** | **Evidencing quotes** |
| --- | --- |
| Theme 1: Moderation as a pillar of community safety  This theme relates to the importance of moderation in online spaces, where such importance is ultimately related to moderation being critical in creating a safe space and providing the foundation for peer support to occur | “I think, because there was quite an emphasis on safety and it being a very safe platform, that it felt more like, less extreme [than other platforms]. Which is, definitely, that's a good thing.” – Moderator 3  “I think my main concern about anything to do with the moderation was medical misinformation and escalating within a thread that we wouldn't be able to monitor within the 24 hours, or however long it would take for the next moderator to come on.” – Moderator 2  “I think that's the thing, is you need people there. If someone starts saying, how many tablets do I need to take to kill myself, you need to act. You can't ignore that. That is serious stuff. And sometimes people do.” – Community member 6, acknowledger |
| Theme 2: The moderation blueprint  This theme relates to everything laying the foundations for moderation to occur - the blueprint of the policies, community principles, moderator guidance, how well these worked, how they resonated with the community, and the importance of this. | “…Don't remember it, which is probably a good thing, because then I probably thought, oh, yes, all of that's fair, of course, it’s how it should be. If I thought it was excessive, then I probably would have remembered it.” – Community member 18, acknowledger  “[I] got the sense that maybe some people wanted to share more personal information that would have maybe helped them to feel more connected” – Moderator 1  “…A really complicated illness that you really don't want to share a lot about. You might have wanted to join it just to read through things. So, yes, for me, it's good. But I wouldn't mind too much if it wasn't anonymous, personally.” – Community member 15, reader  “Some way of making it clear which comments need you to look at and which are less important, I think would be useful. But we got pretty close to it with the time stamps, but I'm sure it could be made even easier” – Moderator 4  “[…] Somebody who wasn't very happy about their personal identifying information being taken out of their posts. I wonder if creating that sense of community for some people is easier, if you are able to share a little bit more information about who you are and what part of the country you live in, and things like that.” – Moderator 1.  “They added timestamps on the last comment, which made it easier, because then you could see if it was within 24 hours, which did improve it quite a lot” – Moderator 4 |
| Theme 3: The moderation threshold This theme relates to the moderator’s experience of navigating the balance of intervening and allowing a situation to play out. Moderators and users have shared concerns about getting the threshold wrong and the impacts it can have on the community. | “Again with, you know, because it's online and you can't obviously see them, see, people, you don't know how different people's communication styles are.” – Moderator 2  “Later in the process, we were doing a lot more of that, I think, of just keeping an eye. And it's reassuring that you're not forgetting about it, you're seeing where it goes, and generally, it would go in a more positive direction without us intervening.” – Moderator 1  “And I wanted to , we were doing a lot more of that, I think, of just keeping an eye. And it's reassuring that you're not forgetting about it, you're seeing where it goes, and generally, it would go in a o. And I’ll certainly be perceived, in a certain way, by users, as a moderator. And so, I held myself back quite a lot” – Moderator 1  “I think having people who aren't healthcare professionals, who are more coming from the perspective of that lived experience might help to get a bit more balance there, possibly, in terms of, I guess, the psychological distress part of it.” – Moderator 2  “I find very heavy moderation tends to stagnate conversation, and it tends to make people more likely to argue” – Community member 6, acknowledger  “It’s always difficult with moderation, because you don't want to censor people, but again, the safety has to be the priority.” – Community member 17, acknowledger  “I think it got to the point where, I was trying to do as little as possible in the moderation sessions, and I had a very high threshold for when I thought that I might need to do anything. I think in total, I only did two or three things in the entire three months, because it was mostly just a case of letting it play out” – Moderator 4  “I think it's a really tricky balance between moderating for misinformation and making sure that it's, I think safe is the wrong word, but I guess, that it's helpful. But I feel within the community of people with long-term health conditions, that's a really big topic of debate and conversation.” – Moderator 5  “I probably more went on the side of thinking it should be quite light touch, and worried that over moderation might stifle communication in the community. I think I was quite, maybe, more light touch than others. I'm not sure. I guess we probably all had our own beliefs and concerns and worries that might put us in different parts of the spectrum of how we moderated.” – Moderator 3  “[…] No one really wants to feel like they're being watched. So, good that, I think, it was a nice balance between moderating and then also participating, which I think was quite welcoming.” – Community member 30, content creator |
| Theme four: Self-moderation is the (achievable) dream.  This theme relates to the shared view that in an ideal community, self-moderation would occur. Self-moderation may be better received than from moderators and enhances ownership over the community. We experienced the early inclining’s of self-moderation, but ultimately more time and a stronger sense of community is needed to lay the foundation for this. | “I didn't see anything that caused me any concern. If I had have done, then I would have reported that to them anyway, but as I say, I was just conscious that I didn't share anything in my own posts that could identify who I was.” – Community member 6, acknowledger  “And someone did mention, like, please don't use acronyms because they couldn’t understand what they were talking about. And then they did change them. But that was the group policing itself, which was quite nice.” – Community member 19, acknowledger  “I felt like the self-moderation was much more powerful in terms of influencing and changing opinion, or getting people to think about things in a different way, because it came from a peer. And so, I think that was a lot more powerful than us coming in as moderators and saying, oh, no, what you're saying is wrong. Which I felt could shut down conversation.” – Moderator 3  “I think getting the conversation started was sometimes quite good, where it would be a story or a, I don't know, something to stimulate discussion. But again, I think there was a similar thing, wherein if they were the first one to comment on some things, say someone said they were having a rubbish time, and then the engagement person would say, I'm so sorry to hear that. Then it might stop the community supporting each other, because it would be like, oh, yes, that's done.  But actually, probably it’s more powerful to hear that from your peers and maybe sounds a... It's hard to know how things might land, but they might just feel a bit, I don't know. It might not feel genuine, I guess. Even, I'm sure it was. And they don't want to… I just think when things are said from their peers, it's more powerful.” – Moderator 3  “As things progressed, as the site progressed, into something more slicker and better, then I would say that moderators would maybe have a slightly different role.” – Community member 11, content creator  “You've got to protect the people that are using the app. And at the end of the day, if you don't do that, then as a company, that's on you, isn't it, if anything happens. You're safeguarding yourselves and the people that you work with” – Community member 33, never logged in |
| Theme 5: Being part of the moderation team  This theme relates to the value that the moderators felt as a result of being part of the moderation TEAM. The benefits relate to the practicalities of balancing workload, but also extend beyond this to becoming a source of learning toward moderation efficiency, but also the provision of emotional support to one another as the moderation team itself became a form of peer support | “…It felt very open door, in terms of people being approachable. If we had any questions, it felt very safe and supported.” – Moderator 3  “It seemed like a good system, and people could sign up for as much or as little as they wanted to do. That felt fair and it felt like you could flex it to your schedule. So, that worked well.” – Moderator 3  “I found that quite interesting and helpful with the handover document, that there'd be something that you might be a bit concerned about, or a bit unsure of how to respond to, or whether you were overreacting to something or not. And then seeing how other people have commented on the same issue was quite helpful, like learning experience.” – Moderator 2  “It's quite good to sense check if there was something that you were a bit worried about, you could flag it up and then you’d usually get a reply. That was quite helpful.” – Moderator 4 |
