# Supplementary material for: Insights and Recommendations From Moderators and Community Members for Keeping Online Peer Support Safe: Thematic Analysis
Source: J Med Internet Res. 2026 Mar 12;28:e81943. doi: 10.2196/81943 (PMC13022541; doi:10.2196/81943)
Supplement: Multimedia Appendix 5 [file jmir_v28i1e81943_app5.docx]

# Appendix 5. Comparison of moderator comments on CommonGround to moderation guidance.

| **Moderation topic** | **Extract of guidance from mod guidance or example response** | **Responses by moderators on CommonGround** |
| --- | --- | --- |
| Medical advice | While we appreciate you participating on CommonGround, we ask that you don’t directly recommend treatments or medications for other members. Please remember that everyone is different – what might work for one person might not work for someone else. We encourage everyone to discuss treatment and medication options with their GP.  It is great to see our community members sharing their experiences of different treatments and medications. Please remember that decisions about someone’s medical care is between themselves and their medical team/GP only. Please avoid promoting specific treatments/medications. | Hi @username, sorry to hear that you have been having a flare-up and we hope you are feeling better soon. On CommonGround we cannot offer specific medical advice. We recommend that you speak to a healthcare professional about your concerns, such as contacting your GP or calling 111 for advice. If things are getting worse and you are very unwell, you should seek emergency support from A&E.  Hi @username, sorry to hear that you have been experiencing near-fainting and falling - this must be scary. We recommend that you speak to a healthcare professional about your concerns, such as contacting your GP or calling 111 for advice if things become worse. If you become unsafe and experience any injuries after falling, you should seek emergency support from A&E. On CommonGround we cannot offer specific medical advice - this includes information about diagnostic tests but can see @username has linked to further reading and resources about PoTs which you may find helpful. Thanks, @username for sharing this with us!  Hi @username, sorry to hear you have been unwell with Covid. Glad that you are feeling better and we hope you continue to improve. It sounds like you did the right thing in contacting 111 and getting medical advice locally. We cannot give specific medical advice on CommonGround but there are some links on this NHS website that might be helpful for information: [URL]. This tool can also be helpful in deciding if other health information can be trusted: [URL] .  I am sure other members of the community will respond with their experiences soon.  Take care, The CommonGround Team  Hi @username, thanks for sharing information from your other communities. If members of the community wish to find out more about treatment options for Crohn's they may find this website helpful: [URL]. We recommend that everyone discusses the treatment options with their healthcare teams and in our "Resources" section we have some information to help with these discussions. Take care, The CommonGround Team |
| Emotional distress | Hi @username, it is great to see you sharing how you are feeling with the community. We hope that our community members can offer you advice and support soon, as we are sure that others have had similar experiences. If you need further help, you might find useful information on our crisis and support pages.  Take care, the CommonGround team  Hi @username, thank you for sharing how you are feeling - we are pleased that other community members have offered you advice and support. Remember we have some resources that might help you if you are having a difficult time. You could try talking to someone you trust, like a friend or family member (here you can find some tips on how to [talk about your mental health with family and friends.)](https://www.mind.org.uk/information-support/guides-to-support-and-services/seeking-help-for-a-mental-health-problem/talking-to-friends-family/)  We know that this can be hard to do, so you can also call the Samaritans anonymously on 116 123 for free (24/7 365 days a year). If you would rather text, SHOUT offers 24/7 mental health support all year round – simply text SHOUT to 85258.  Take care, the CommonGround team | Hello @username, sorry to hear you have been feeling out of sorts. Thank you for sharing how you are feeling so honestly with the community, this can benefit you and other members of the community. It is great to see some of the community already offering you support, and we hope that everyone can continue to share advice and listen to one another. If at any time you need more help or support, you can find out how to access this on our crisis and support pages (you can click on the banner at the top of the page to access this).  Take care, the CommonGround team  Thank you @username for sharing what sounds like a very distressing time in your life. As @username and @username have said, we're pleased things ended up improving for you from a mental health standpoint. I'd like to take the opportunity to remind any members of the community who may be feeling similarly, in crisis, or worried about someone else, that support and crisis resources can be found in the tab at the top of the page. In there, you will see ways to find one-to-one support or access more information. Take care, the CommonGround team.  Hi @username, thank you for posting and sharing how you are feeling. It is great to see the community offering support. If at any time you need more help or support, we have some Resources that might help you in the "Support & Crisis" section. We know that taking the step to getting further help can be difficult, so talking with someone we trust can be a good start, such as friends, family or your GP. Alternatively, if you wanted to talk to someone anonymously and confidentially, then you could call the Samaritans on 116 123 or text Shout to 85258. Both services provide one-to-one support that is free and available 24/7 every day. Take care, the CommonGround team  Hi @username, sorry to hear you are feeling this way. We're glad to see the community offering support but if at any time you need more help or support, we have some resources that might help you in the "Support & Crisis" section. We know that taking the step to getting further help can be difficult, so talking with someone we trust can be a good start, such as friends, family or your GP. Alternatively, if you wanted to talk to someone anonymously and confidentially, then you could call the Samaritans on 116 123 or text Shout to 85258. Both services provide one-to-one support that is free and available 24/7 every day. Take care, the CommonGround team  Hi @username, thank you for sharing your experiences and your feelings with the community. I am sure that members of the community will be in touch soon to offer their support and share their experiences. In the meantime, there are some helpful materials in our "Resources" section that might be helpful for you- you can filter the resources based on different topics. If you ever feel you need more support there are some options for accessing this in our "Support &Crisis" pages. Take care, The CommonGround Team. |
